# Weighted stochastic block model

**DOI:** 10.1007/s10260-021-00590-6

**Published:** 2021-09-13

**Authors:** Tin Lok James Ng, Thomas Brendan Murphy

**Affiliations:** 1 School of Computer Science and Statistics, Trinity College Dublin, Dublin, Ireland; 2 School of Mathematics and Statistics, University College Dublin, Dublin, Ireland

**Keywords:** Weighted stochastic block model, Variational estimators, Maximum likelihood estimators, Consistency, Model selection

## Abstract

We propose a weighted stochastic block model (WSBM) which extends the stochastic block model to the important case in which edges are weighted. We address the parameter estimation of the WSBM by use of maximum likelihood and variational approaches, and establish the consistency of these estimators. The problem of choosing the number of classes in a WSBM is addressed. The proposed model is applied to simulated data and an illustrative data set.

## Introduction

Networks are used in many scientific disciplines to represent interactions among objects of interest. For example, in the social sciences, a network typically represents social ties between actors. In biological sciences, a network can represent interactions between proteins.

The stochastic block model (SBM) (Holland et al. 1983; Snijders and Nowicki 1997) is a popular generative model which partitions vertices into latent classes. Conditional on the latent class allocations, the connection probability between two vertices depends only on the latent classes to which the two vertices belong. Many extensions of the class SBM have been proposed which include the degree correlated SBM (Karrer and Newman 2011; Peng and Carvalho 2016), mixed membership SBM (Airoldi et al. 2008) and overlapping SBM (Latouche et al. 2011).

The SBM and many of its variants are usually restricted to Bernoulli networks. However, many binary networks are produced after applying a threshold to a weighted relationship (Ghasemian et al. 2016) which results in the loss of potentially valuable information. Although most of the literature has focused on binary networks, there is a growing interest in weighted graphs (Barrat et al. 2004; Newman 2004; Peixoto 2018).

In particular, a number of clustering methods have been proposed for weighted graphs including algorithm based and model based methods. Algorithm based methods for clustering of weighted graphs can be further divided into two classes: algorithms which do not explicitly optimize any criteria (Pons and Latapy 2005; von Luxburg 2007) and those directly optimize a criterion (Clauset et al. 2004; Stouffer and Bascompte 2011). Model based methods (Mariadassou et al. 2010; Aicher et al. 2013, 2015; Ludkin 2020) attempt to take into account the random variability in the data. A recent review of graph clustering methods is given by (Leger et al. 2014).

Mariadassou et al. (2010) presents a Poisson mixture random graph model for integer valued networks and proposes a variational inference approach for parameter estimation. The model can account for covariates via a regression model. In Zanghi et al. (2010), a mixture modelling framework is considered for random graphs with discrete or continuous edges. In particular, the edge distribution is assumed to follow an exponential family distribution. Aicher et al. (2013) proposed a general class of weighted stochastic block model for dense graphs where edge weights are assumed to be generated according to an exponential family distribution. In particular, their construction produces complete graphs, in which every pair of vertices is connected by some real-valued weight. Since most real-world networks are sparse, the constructed model cannot be applied directly. To address this shortcoming, Aicher et al. (2015) extends the work of Aicher et al. (2013) and models the edge existence using a Bernoulli distribution and the edge weights using an exponential family distribution. The contributions of edge-existence distribution and edge-weight distribution in the likelihood function are then combined via a simple tuning parameter. However, their construction does not result in a generative model and it is not obvious how to simulate network observations from the proposed model. More recently, Ludkin (2020) presents a generalization of the SBM which allows artbitrary edge weight distributions and proposes a reversible jump Markov chain Monte Carlo sampler for estimating the parameters and the number of blocks. However, the use of continuous probability distribution to model the edge weights implies that the resulting graph is complete whereby every edge is present. This assumption is unrealistic for many applications whereby a certain proportion of the real-valued edges is 0. Haj et al. (2020) presents a binomial SBM for weighted graphs and proposes a variational expectation maximization algorithm for parameter estimation.

In this paper, we propose a weighted Stochastic Block model (WSBM) with gamma weights which aims to capture the information of weights directly using a generative model. Both maximum likelihood estimation and variational methods are considered for parameter estimation where consistency results are derived. We also address the problem of choosing the number of classes using the Integrated Completed Likelihood (ICL) criteria (Biernacki et al. 2000). The proposed models and inference methodology are applied to an illustrative data set.

## Model specification

In this section, we present the weighted stochastic block model in detail and introduce the main notations and assumptions.

We let $\varOmega = ({\mathcal {V}}, {\mathcal {X}}, {\mathcal {Y}})$ denote the set of directed weighted random graphs where ${\mathcal {V}} = \mathbb {N}$ is the set of countable vertices, ${{\mathcal {X}}} = \{ 0, 1 \}^{\mathbb {N} \times \mathbb {N}}$ is the set of edge-existence adjacency matrix, and ${\mathcal {Y}} = \mathbb {R}_{+}^{ \mathbb {N} \times \mathbb {N} }$ is the set of weighted adjacency matrix. Given a random adjacency matrix $X = \{ X_{ij} \}_{i,j \in \mathbb {N}}$, $X_{ij}=1$ if an edge exists from vertex *i* to vertex *j* and $X_{ij}=0$ otherwise. The associated weighted random adjacency matrix is given by: for $i \ne j$, if $X_{ij}=1$, $Y_{ij} > 0$, and $Y_{ij}=0$ otherwise. Let $\mathbb {P}$ be a probability measure on $\varOmega$.

### Generative model

We now describe the procedure of generating a sample of random graph (*V*, *X*, *Y*) with *n* vertices from $\varOmega$.

- Let $Z_{[n]}=(Z_{1}, \ldots , Z_{n})$ be the vector of latent block allocations for the vertices, and set $\theta =(\theta _{1}, \ldots , \theta _{Q})$ with $\sum _{q} \theta _{q}=1$. For each vertex $v_{i}$, draw its block label $Z_{i} \in \{1, \ldots , Q\}$ from a multinomial distribution
  $$\begin{aligned} Z_{i} \sim {{\mathcal {M}}}(1; \theta _{1}, \ldots , \theta _{Q}) \text{. } \end{aligned}$$
- Let $\pi = (\pi _{ql})_{q,l=1}^{Q}$ be a $Q \times Q$ matrix with entries in [0, 1]. Conditional on the block allocations $Z_{[n]}$, the entries $X_{ij}$ for $i \ne j$ of the edge-existence adjacency matrix $X_{[n]}$ is generated from independently a Bernoulli distribution
  $$\begin{aligned} X_{ij} | Z_{i}=q, Z_{j}= l \sim {{\mathcal {B}}}(\pi _{ql}) \text{. } \end{aligned}$$
- Let $\alpha = (\alpha _{ql})_{q,l=1}^{Q}$ and $\beta =(\beta _{ql})_{q,l=1}^{Q}$ be $Q \times Q$ matrices with entries taking values in the positive reals. Conditional on the latent block allocations $Z_{[n]}$ and edge-existence adjacency matrix *X*, the weighted adjacency matrix $Y_{[n]}$ is generated independently from
  $$\begin{aligned} Y_{ij} | X_{ij} = 1, Z_{i} = q, Z_{j} = l\sim & {} \text{ Ga }(\alpha _{ql}, \beta _{ql}) \text{, } \\ Y_{ij} | X_{ij} = 0, Z_{i} = q, Z_{j} = l\sim & {} \delta _{\{0\}} \text{, } \end{aligned}$$
  where $\text{ Ga }(\cdot ,\cdot )$ denotes the gamma distribution and $\delta _{\{\cdot \}}$ is the Dirac delta function.

The generative framework described above is a straightforward extension of the binary stochastic block model whereby a positive weight is generated according to a gamma distribution for each edge. In particular, (*X*, *Z*) is a realization of the binary directed SBM. The gamma distribution is chosen due to its flexibility in the sense that, depending on the value of its shape parameter, it can represent distributions of different shapes.

The log-likelihood of the observations $X_{[n]}$ and $Y_{[n]}$ is given by

1 $$\begin{aligned} {{\mathcal {L}}}_{2}(Y_{[n]}, X_{[n]}; \theta , \pi , \alpha , \beta ) = \log \Big ( \sum _{z_{[n]}} e^{ {{\mathcal {L}}}_{1}(Y_{[n]}, X_{[n]}; z_{[n]}, \pi , \alpha , \beta ) } \mathbb {P}\{ Z_{[n]} = z_{[n]} \} \Big ) , \end{aligned}$$

where the sum is over all possible latent block allocations, $\mathbb {P} \{ Z_{[n]} = z_{[n]} \} = \prod _{i=1}^{n} \theta _{z_{i}}$ is the probability of latent block allocation $z_{[n]}$, and

2 $$\begin{aligned}&{{\mathcal {L}}}_{1}(Y_{[n]}, X_{[n]}; z_{[n]}, \pi , \alpha , \beta )&\nonumber \\&\quad = \sum _{i \ne j} \Big [ X_{ij} \big \{ \log \pi _{z_{i}, z_{j}} + \log f(Y_{ij}; \alpha _{z_{i},z_{j}}, \beta _{z_{i},z_{j}} ) \big \} + (1-X_{ij}) \log (1-\pi _{z_{i},z_{j}}) \Big ]&\nonumber \\&\quad = \sum _{i \ne j} \Big \{X_{ij} \log \pi _{z_{i}, z_{j}} + (1-X_{ij}) \log (1-\pi _{z_{i},z_{j}}) \Big \} \nonumber \\&\qquad + X_{ij} \Big \{ \alpha _{z_{i},z_{j}} \log \beta _{z_{i},z_{j}} + (\alpha _{z_{i},z_{j}} -1) \log Y_{ij} - \beta _{z_{i},z_{j}} Y_{ij} - \log \varGamma (\alpha _{z_{i},z_{j}}) \Big \}&\end{aligned}$$

is the complete data log-likelihood, where $f(\cdot ;a,b)$ is the gamma probability density function with shape parameter *a* and rate parameter *b*,

### Assumptions

We present several assumptions needed for identifiability and consistency of maximum likelihood estimates. The following four assumptions were presented in Celisse et al. (2012) and are needed in this paper.

#### Assumption 1

For every $q \ne q^{'}$, there exists $l \in \{1, \ldots , Q\}$ such that

$$\begin{aligned} \pi _{q,l} \ne \pi _{q^{'},l} \text{ or } \pi _{l,q} \ne \pi _{l,q^{'}} . \end{aligned}$$

#### Assumption 2

There exists $\zeta \in (0,1)$ such that for all $(q,l) \in \{1, \ldots , Q \}^{2}$

$$\begin{aligned} \pi _{ql} \in [\zeta , 1-\zeta ] . \end{aligned}$$

#### Assumption 3

There exists $0< \gamma < 1/Q$ such that for all $q \in \{1, \ldots , Q\}$,

$$\begin{aligned} \theta _{q} \in [\gamma , 1-\gamma ] . \end{aligned}$$

#### Assumption 4

There exists $0< \gamma < 1/Q$ and $n_{0} \in \mathbb {N}^{*}$ such that for all $q \in \{1, \ldots , Q\}$, for all $n \ge n_{0}$,

$$\begin{aligned} \frac{ N_{q}(z^{*}_{[n]} ) }{n } \ge \gamma , \end{aligned}$$

where $N_{q}(z^{*}_{[n]} ) = | \{ 1 \le i \le n : z^{*}_{i} = q |$ and $z^{*}_{[n]}$ is any realized block allocation under the WSBM.

(A1) requires that no two classes have the same connectivity probabilities. If this assumption is violated, the resulting model has too many classes and is non-identifiable. (A2) requires that the connectivity probability between any two classes strictly lies within a closed subset of the unit interval. Note that this assumption is slightly more restrictive compared to assumption 2 of Celisse et al. (2012) in that we do not consider the boundary cases where $\pi _{q,l} \in \{0,1\}$. The boundary cases require special treatment and are not pursued in this paper. (A3) ensures that no class is empty with high probability while (A4) is the empirical version of (A3). We note that (A4) is satisfied asymptotically under the generative framework in Sect. 2.1 since the block allocations are generated according to a multinomial distribution.

In addition to the four assumptions above, we also have the following constraints on the gamma parameters.

#### Assumption 5

For every $q \ne q^{'}$, there exists $l \in \{1, \ldots , Q \}$ such that

$$\begin{aligned} ( \alpha _{q,l}, \beta _{q,l}) \ne (\alpha _{q^{'},l}, \beta _{q^{'},l}) \text{ or } (\alpha _{l,q}, \beta _{l, q}) \ne (\alpha _{l,q^{'}},\beta _{l,q^{'}}) \end{aligned}$$

(A5) requires that no two classes have the same weight distribution. This assumption is the exact counterpart of (A1).

The log-likelihood function (1) contains degeneracies that prevent the direct estimation of parameters $\theta , \pi , \alpha , \beta$. To see this, we note that the probability density function of a gamma distribution $\text{ Ga }(a,b)$ is given by

$$\begin{aligned} f(y;a,b) = \frac{ y^{a-1} \exp (-b y) b^{a} }{ \varGamma (a) } \text{. } \end{aligned}$$

By Stirling’s formula, we have

$$\begin{aligned} \varGamma (a) = \sqrt{2 \pi } a^{a-1/2} \exp (-a) (1 + {{\mathcal {O}}}(a^{-1}) ) \text{. } \end{aligned}$$

Setting $$y = ab$$,

$$\begin{aligned} f(y;a,b) = \frac{ y^{a-1} \exp (-a) (a/y)^{a} }{ \varGamma (a) } = \frac{1}{ \sqrt{2 \pi } y} \frac{ \sqrt{a} }{ 1 + {{\mathcal {O}}}(a^{-1}) } \text{. } \end{aligned}$$

Therefore, letting $a \rightarrow \infty$ while keeping $ab=y$, we have $f(y;a,b) \rightarrow \infty$. One can therefore show that the log-likelihood function is unbounded above. To avoid likelihood degeneracy, we compactify the parameter space. That is, we restrict the parameter space to a compact subset which contains the true paraemters. Therefore, we have the following assumption.

#### Assumption 6

There exists $0< \alpha _{c}< \alpha _{C} < \infty$ and $0< \beta _{c}< \beta _{C} < \infty$ such that for all $(q,l) \in \{ 1, \ldots , Q \}$,

$$\begin{aligned} \alpha _{c} \le \alpha _{ql} \le \alpha _{C} \text{, } \beta _{c} \le \beta _{ql} \le \beta _{C} \text{. } \end{aligned}$$

With this assumption, it is easy to see that the log-likelihood function is bounded for any sample size.

### Identifiability

Sufficient conditions for identifiability of binary SBM with two classes have been first obtained by Allman et al. (2009). Celisse et al. (2012) show that the SBM parameters are identifiable up to a permutation of class labels under the conditions that $\pi \theta$ has distinct coordinates and $n \ge 2Q$. The condition on $\pi \theta$ is mild since the set of vectors violating this assumption has Lebesgue measure 0. The identifiability of weighted SBM is more challenging where the only known result (Section 4 of Allman et al. (2011)) requires all entries of $(\pi , \alpha , \beta )$ to be distinct. We note that the assumptions in the previous section are not necessarily sufficient but are necessary to ensure that the identifiability of the parameters.

## Asymptotic recovery of class labels

We study the posterior probability distribution of the class labels $Z_{[n]}$ given the random adjacency matrix $X_{[n]}$ and weight matrix $Y_{[n]}$, which is denoted by $\mathbb {P}(Z_{[n]}|X_{[n]},Y_{[n]})$. Since $X_{[n]}$ and $Y_{[n]}$ are random, $\mathbb {P}(Z_{[n]}|X_{[n]},Y_{[n]})$ is also random.

Let $P^{*}(X_{[n]},Y_{[n]}) := \mathbb {P}(X_{[n]},Y_{[n]}|Z_{[n]}=z_{[n]}^{*})$ be the true conditional distribution of $(X_{[n]},Y_{[n]})$ which depends on the true parameters $(\theta ^{*},\pi ^{*},\alpha ^{*},\beta ^{*})$. We study the convergence rate of $\mathbb {P}(Z_{[n]}|X_{[n]},Y_{[n]})$ towards 1 with respect to $P^{*}$.

The matrices $\pi , \alpha , \beta$ are permutation-invariant if one permutes both its rows and columns according to some permutation $\sigma : \{1,\ldots ,Q\} \rightarrow \{1,\ldots ,Q\}$. Let $\pi ^{\sigma }, \alpha ^{\sigma }, \beta ^{\sigma }$ be the matrices defined by

$$\begin{aligned} \pi ^{\sigma }_{ql} = \pi _{\sigma (q),\sigma (l)} \text{, } \alpha ^{\sigma }_{ql} =\alpha _{\sigma (q), \sigma (l)} \text{, } \beta ^{\sigma }_{ql} = \beta _{\sigma (q), \sigma (l)} \end{aligned}$$

and define the set

$$\begin{aligned} \varSigma = \{ \sigma : \{1,\ldots ,Q\} \rightarrow \{1,\ldots ,Q\} | \pi ^{\sigma } = \pi , \alpha ^{\sigma } = \alpha , \beta ^{\sigma }=\beta \} \text{. } \end{aligned}$$

Two vectors of class labels *z* and $z^{'}$ are equivalent if there exists $\sigma \in \varSigma$ such that $z^{'}_{i} = \sigma (z_{i}) \text{, }$ for all *i*. We let [*z*] denote the equivalence class of *z* and will omit the square-brackets in the equivalence class notation as long as no confusion arises.

The following result extends Theorem 3.1 of Celisse et al. (2012) to the case of WSBM.

### **Theorem 1**

*Under assumptions (A1)–(A6), for every* $t>0$,

$$\begin{aligned} P^{*} \Bigg [ \sum _{ [z_{[n]}] \ne [z^{*}_{[n]}] } \frac{ \mathbb {P}( [ Z_{[n]} ] = [ z_{[n]} ] | X_{[n]}, Y_{[n]} )}{ \mathbb {P}( [ Z_{[n]} ] = [ z^{*}_{[n]} ] | X_{[n]}, Y_{[n]} ) } > t \Bigg ] = {{\mathcal {O}}}(n e^{- \kappa n}) \end{aligned}$$

*uniformly with respect to* $z^{*}$, *and for some* $\kappa > 0$ *depending only on* $\pi ^{*}, \alpha ^{*}, \beta ^{*}$ *but not on* $z^{*}$. Here $z^{*} = (z_i^{*})_{i=1}^{\infty }$ *with* $z_i^{*} \in \{1, \ldots , Q\}$. *Furthermore*, $P^{*}$ *can be replaced by* $\mathbb {P}$ *under assumptions (A1)–(A3) and (A5)–(A6)*.

## Maximum likelihood estimation of WSBM parameters

For the binary SBM, consistency of parameter estimation have been shown for profile likelihood maximization (Bickel and Chen 2009), spectral clustering method (Rohe et al. 2011), method of moments approach (Bickel et al. 2011), method based on empirical degrees (Channarond et al. 2012), and others (Choi et al. 2012). Consistency of both maximum likelihood estimation and variational approximation method are established in Celisse et al. (2012) and Bickel et al. (2013) where asymptotic normality is also established in Bickel et al. (2013). Abbe (2018) reviews recent development in the stochastic block model and community detections.

Ambroise and Matias (2012) proposes a general class of sparse and weighted SBM where the edge distribution may exhibit any parametric form and studies the consistency and convergence rates of various estimators considered in their paper. However, their model requires the edge existence parameter to be constant across the graph, that is, $\pi _{ql} = \pi , \quad$ for all *q*, *l*, or $\pi _{ql}$ can be modelled as $\pi _{ql} = a_1 I_{q =l} + a_2 I_{q \ne l}$ where $I_{\cdot }$ is the indicator function and $a_1 \ne a_2$. Furthermore, they also assume that conditional on the block assignments, the edge weight $Y_{ij}|X_{i}=q, X_{j}=l$ is modelled using a parametric distribution with a single parameter $\theta _{ql}$. They further impose the restriction that

$$\begin{aligned} \theta _{ql} = {\left\{ \begin{array}{ll} \theta _{in} \quad \text{ if } q = l, \\ \theta _{out} \quad \text{ if } q \ne l. \end{array}\right. } \end{aligned}$$

These assumptions are more restrictive than those imposed in this paper. Jog and Loh (2015) studies the problem of characterizing the boundary between success and failure of MLE when edge weights are drawn from discrete distributions. More recently, Brault et al. (2020) studies the consistency and asymptotic normality of the MLE and variational estimators for the latent block model which is a generalization of the SBM. However, the model considered in Brault et al. (2020) is restricted to the dense setting and requires the observations in the data matrix to be modelled by univariate exponential family distributions.

This section addresses the consistency of the MLE of WSBM. In particular, we extend the results obtained in the pioneering paper of Celisse et al. (2012) to the case of weighted graphs. Our proof closely follows the proof of consistency of the MLE in Celisse et al. (2012). The MLE consistency proof of $( \pi , \alpha , \beta )$ and $\theta$ require different treatments since there are $n(n-1)$ edges but only *n* vertices. The following result established the MLE consistency of $(\pi , \alpha , \beta )$.

### **Theorem 2**

*Assume that assumptions (A1), (A2), (A3), (A5), (A6) hold. Let us define the MLE of* $( \theta ^{*}, \pi ^{*}, \alpha ^{*}, \beta ^{*})$ *by*

$$\begin{aligned} (\hat{ \varvec{ \theta } }, \hat{ \pi }, \hat{ \alpha }, \hat{\beta }) := \arg \max _{\theta , \pi , \alpha , \beta } {{\mathcal {L}}}_{2}(Y_{[n]}, X_{[n]}; \theta , \pi , \alpha , \beta ) . \end{aligned}$$

*Then for any metric* $d(\cdot ,\cdot )$ *on* $(\pi , \alpha , \beta )$,

$$\begin{aligned} d( ( \hat{\pi }, \hat{\alpha }, \hat{\beta }), ( \pi ^{*}, \alpha ^{*}, \beta ^{*}) ) \xrightarrow [ n \rightarrow \infty ]{ \mathbb {P} } 0 . \end{aligned}$$

Under additional assumption on the rate of convergence of the estimators $(\hat{\pi }, \hat{\alpha }, \hat{\beta })$ of $(\pi , \alpha , \beta )$, consistency of $\hat{\theta }$ can be established.

### **Theorem 3**

*Let* $(\hat{\theta }, \hat{\pi }, \hat{\alpha }, \hat{\beta } )$ *denote the MLE of* $(\theta ^{*}, \pi ^{*}, \alpha ^{*}, \beta ^{*})$ *and assume that* $|| \hat{\pi } - \pi ^{*} ||_{\infty } = o_{\mathbb {P}}( \sqrt{\log n}/n )$, $|| \hat{\alpha } - \alpha ^{*} ||_{\infty } = o_{\mathbb {P}}( \sqrt{\log n}/n )$, *and* $|| \hat{\beta } - \beta ^{*} ||_{\infty } = o_{\mathbb {P}}( \sqrt{\log n}/n )$, *then*

$$\begin{aligned} d( \hat{\theta }, \theta ^{*}) \xrightarrow [n \rightarrow \infty ]{\mathbb {P}} 0 \end{aligned}$$

*for any metric* *d* *in* $\mathbb {R}^{Q}$.

## Variational estimators

Direct maximization of the log-likelihood function is intractable except for very small graphs since it involves a sum over $Q^{n}$ terms. In practice, approximate algorithms such as Markov Chain Monte Carlo (MCMC) and variational inference algorithms are often used for parameter inference. For the SBM, both MCMC and variational inference approaches have been proposed (Snijders and Nowicki 1997; Daudin et al. 2008). Variational inference algorithms have also been developed for mixed membership SBM (Airoldi et al. 2008), overlapping SBM (Latouche et al. 2011), and the weighted SBM proposed in Aicher et al. (2013). This section develops a variational inference algorithm for the WSBM which can be considered a natural extension of the algorithm proposed in Daudin et al. (2008) for the SBM.

The variational method consists in approximating $P(Z_{[n]}= \cdot | X_{[n]}, Y_{[n]})$ by a product of *n* multinomial distributions. Let ${{\mathcal {D}}}_{n}$ denote a set of product multinomial distributions

$$\begin{aligned} {{\mathcal {D}}}_{n} = \Big \{ D_{\tau _{[n]}} = \prod _{i=1}^{n} {{\mathcal {M}}}(1; \tau _{i,1}, \ldots , \tau _{i,Q}) | \tau _{[n]} \in {{\mathcal {S}}}_{n} \Big \} \end{aligned}$$

where

$$\begin{aligned} {{\mathcal {S}}}_{n} = \Big \{ \tau _{[n]} = (\tau _{1}, \ldots , \tau _{n}) \in ([0,1]^{Q})^{n} | \text{ for } \text{ all } i, \tau _{i} = (\tau _{i,1}, \ldots , \tau _{i,Q}), \sum _{q=1}^{Q} \tau _{i,q} = 1 \Big \} \text{. } \end{aligned}$$

For any $D_{\tau _{[n]}} \in {{\mathcal {D}}}_{n}$, the variational log-likelihood is defined by

$$\begin{aligned} {{\mathcal {J}}}(Y_{[n]},X_{[n]}; \tau _{[n]}, \theta , \pi , \alpha , \beta ) = {{\mathcal {L}}}_{2}(Y_{[n]}, X_{[n]}; \theta , \pi , \alpha , \beta ) - \mathbb {KL}(D_{\tau _{[n]}}, P(\cdot |X_{[n]}, Y_{[n]})) \text{. } \end{aligned}$$

Here $\mathbb {KL}(\cdot ,\cdot )$ denotes the Kullback-Leibler divergence between two probability distributions, which is nonnegative. Therefore, ${{\mathcal {J}}}$ provides a lower bound on the log-likelihood function. We have that

$$\begin{aligned} {{\mathcal {J}}}(Y_{[n]},X_{[n]}; \tau _{[n]}, \theta , \pi , \alpha , \beta ) = \sum _{i \ne j} \sum _{q,l} \tau _{i,q} \tau _{jl} \Big ( \log \text{ b }(X_{ij}; \pi _{ql}) + X_{ij} \log f(Y_{ij}; \alpha _{ql}, \beta _{ql}) \Big ) \\ - \sum _{i} \sum _{q} \tau _{iq} ( \log \tau _{iq} - \log \theta _{q} ) \end{aligned}$$

where $\text{ b }(\cdot ;p)$ denotes the probability mass function of a Bernoulli distribution with parameter *p*, and recall that $f(\cdot ;\alpha _{ql},\beta _{ql})$ denotes the density function of a gamma distribution $\text{ Ga }(\alpha _{ql},\beta _{ql})$.

The variational algorithm works by iteratively maximizing the lower bound ${{\mathcal {J}}}$ with respect to the approximating distribution $D_{\tau _{[n]}}$, and estimating the model parameters. Maximization of ${{\mathcal {J}}}$ with respect to $D_{\tau _{[n]}}$ consists of solving

$$\begin{aligned} \hat{\tau }_{[n]} := \text{ argmax}_{\tau _{[n]}} {\mathcal {J}}(Y_{[n]},X_{[n]}; \tau _{[n]}, \theta , \pi , \alpha , \beta ) \text{, } \end{aligned}$$

where $\theta , \pi , \alpha , \beta$ can be replaced by plug-in estimates. This has a closed-form solution given by

3 $$\begin{aligned} \hat{\tau }_{iq} \propto \theta _{q} \prod _{j \ne i} \prod _{l} \text{ b }(X_{ij};\pi _{ql})^{\hat{\tau }_{jl}} f(Y_{ij}; \alpha _{ql}, \beta _{ql})^{ \hat{\tau }_{jl} X_{ij} } \text{. } \end{aligned}$$

Conditional on $\hat{\tau }_{[n]}$, the variational estimators of $(\theta , \pi , \alpha , \beta )$ are found by solving,

$$\begin{aligned} (\tilde{\theta }, \tilde{\pi }, \tilde{\alpha }, \tilde{\beta }) = \text{ argmax}_{\theta , \pi , \alpha , \beta } {\mathcal {J}}(Y_{[n]},X_{[n]}; \tau _{[n]}, \theta , \pi , \alpha , \beta ) . \end{aligned}$$

Closed-form updates for $\tilde{\theta }$ and $\tilde{\pi }$ exist and are given by

4 $$\begin{aligned} \tilde{\theta }_{q}&=  \frac{1}{n} \sum _{i} \hat{ \tau }_{iq} \end{aligned}$$

5 $$\begin{aligned} \tilde{\pi }_{ql}&=  \frac{ \sum _{i \ne j} \hat{\tau }_{iq} \hat{\tau }_{jl} X_{ij} }{ \sum _{i \ne j} \hat{\tau }_{iq} \hat{\tau }_{jl} } \text{. } \end{aligned}$$

On the other hand, updates for $\tilde{\alpha }$ and $\tilde{\beta }$ do not have a closed form since the maximum likelihood estimators of the two parameters of a gamma distribution do not have closed forms. However, using the fact that a gamma distribution is a special case of a generalized gamma distribution, Ye and Chen (2017) derived simple closed-form estimators for the two parameters of gamma distribution. The estimators were shown to be strongly consistent and asymptotically normal. For $q,l = 1, \ldots ,Q$, let us define the quantities

$$\begin{aligned} \tilde{W}_{ql}&=  \sum _{i \ne j, X_{ij} = 1} \hat{\tau }_{iq} \hat{\tau }_{jl}\\ \tilde{U}_{ql}&=  \sum _{i \ne j, X_{ij} = 1} \hat{\tau }_{iq}\hat{\tau }_{jl} Y_{ij}\\ \tilde{V}_{ql}&=  \sum _{i \ne j, X_{ij} = 1} \hat{\tau }_{iq}\hat{\tau }_{jl} \log Y_{ij}\\ \tilde{S}_{ql}&=  \sum _{i \ne j, X_{ij} = 1} \hat{\tau }_{iq}\hat{\tau }_{jl} Y_{ij} \log Y_{ij} , \end{aligned}$$

then the updates for $\alpha _{ql}, \beta _{ql}$ are given by

6 $$\begin{aligned} \tilde{\alpha }_{ql}&=  \frac{ \tilde{W}_{ql} \tilde{U}_{ql} }{ \tilde{W}_{ql} \tilde{S}_{ql} - \tilde{V}_{ql} \tilde{U}_{ql} } \end{aligned}$$

7 $$\begin{aligned} \tilde{\beta }_{ql}&=  \frac{ \tilde{W}_{ql}^{2} }{ \tilde{W}_{ql} \tilde{S}_{ql} - \tilde{V}_{ql} \tilde{U}_{ql} } \text{. } \end{aligned}$$

We obtain the variational estimators $(\tilde{\theta }, \tilde{\pi }, \tilde{\alpha }, \tilde{\beta })$ by computing (3), (4), (5), (6), (7) until convergence.

We now address the consistency of the variational estimators derived above. The following two propositions are the counterpart of Theorem 2 and 3 for variational estimators. We omit the proof since they follow similar arguments as the proof of Corollary 4.3 and Theorem 4.4 of Celisse et al. (2012).

### **Proposition 1**

*Assume that assumptions (A1), (A2), (A3), (A5) and (A6), and let* $(\tilde{\theta }, \tilde{\pi }, \tilde{\alpha }, \tilde{\beta } )$ *be the variational estimators defined above. Then for any distance* $d(\cdot ,\cdot )$ on $( \pi , \alpha , \beta )$,

$$\begin{aligned} d( (\tilde{\pi }, \tilde{\alpha }, \tilde{\beta } ), ( \pi ^{*}, \alpha ^{*}, \beta ^{*} ) ) \xrightarrow [ n \rightarrow \infty ]{ \mathbb {P} } 0 . \end{aligned}$$

### **Proposition 2**

*Assume that the variational estimators* $(\tilde{\pi }, \tilde{\alpha }, \tilde{\beta })$ *converge at rate* 1/*n* to $(\pi ^{*}, \alpha ^{*}, \beta ^{*})$, *respectively, and assumptions (A1), (A2), (A3), (A5), (A6) hold. We have*

$$\begin{aligned} d(\tilde{\theta }, \theta ^{*}) \xrightarrow [n \rightarrow \infty ]{\mathbb {P}} 0 , \end{aligned}$$

*where* *d* *denotes any distance between vectors in* $\mathbb {R}^{Q}$.

Note a stronger assumption on the convergence rate 1/*n* of $\tilde{\pi }, \tilde{\alpha }, \tilde{\beta }$ is assumed for Proposition 2 compared to $\sqrt{\log n}/n$ in Theorem 3. The same assumption is also used in Theorem 4.4. of Celisse et al. (2012).

## Choosing the number of classes

In real world applications, the number of classes is typically unknown and needs to be estimated from the data. For the SBM, a number of methods have been developed to determine the number of classes, including log-likelihood ratio statistic (Wang and Bickel 2017), composite likelihood (Saldaña et al. 2017), exact integrated complete data likelihood (Côme and Latouche 2015) and Bayesian framework (Yan 2016) based methods. Model selection for variants of SBM have also been investigated (Latouche et al. 2014).

We apply the integrated classification likelihood (ICL) criterion developed by Biernacki et al. (2000) to choose the number of classes for the WSBM. The ICL is an approximation of the complete-date integrated likelihood. The ICL criterion for SBM have been derived by Daudin et al. (2008) under the assumptions that the prior distribution of $(\theta , \pi )$ factorizes and a non-informative Dirichlet prior on $\theta$. Here we follow the approach of Daudin et al. (2008) to derive an approximate ICL for the WSBM.

Let $m_{Q}$ denote the model with *Q* blocks, the ICL criterion is an approximation of the complete-data integrated likelihood:

$$\begin{aligned}&{{\mathcal {L}}}(Y_{[n]},X_{[n]},Z_{[n]} | m_{Q})&\\&\quad = \int _{ \theta , \pi , \alpha , \beta } {{\mathcal {L}}}(Y_{[n]}, X_{[n]}, Z_{[n]}; \theta , \pi , \alpha , \beta , m_{Q}) g(\theta , \pi , \alpha , \beta ) d \theta d \pi d\alpha d\beta&\text{. } \end{aligned}$$

where $g(\theta , \pi , \alpha , \beta )$ is the prior distribution of the parameters. Assuming a non-informative Jeffreys prior on $\theta$, a Stirling approximation to the gamma function, and finally a BIC approximation to the conditional log-likelihood function, the approximate ICL can be derived. For a model $m_{Q}$ with *Q* blocks, and assuming a non-informative Jeffreys prior $Dir(0.5, \ldots , 0.5)$ on $\theta$, the approximate ICL criterion is:

$$\begin{aligned} ICL(m_{Q})&=  \max _{\theta , \pi , \alpha , \beta } \log L(Y_{[n]}, X_{[n]}, \tilde{Z}_{[n]}; \theta , \pi , \alpha , \beta , m_{Q} ) \\& \quad- \frac{3}{2} (Q(Q+1)) \log n(n-1) - \frac{Q-1}{2} \log n \end{aligned}$$

where $\tilde{Z}_{[n]}$ is the estimate of $Z_{[n]}$. The derivation of above follows exactly the same lines as the proof of Proposition 8 of Daudin et al. (2008).

## Simulation

We validate the theoretical results developed in previous sections by conducting simulation studies. In particular, we investigate how fast parameter estimates of WSBM converge to their true values and the accuracy of posterior block allocations. Additionally, we investigate the performance of ICL in choosing the number of blocks.

### Experiment 1 (two-class model)

For each fixed number of nodes *n*, 50 realizations of WSBM are generated based on the fixed parameter setting given in (8 and 9). The variational inference algorithm derived in Sect. 5 is then applied to estimate the model parameters and class allocations.

We can see from Table 1 that the estimated model parameters converge to their true values as the number of nodes increases while the posterior class assignment is accurate across any number of nodes. Table 2 shows the ICL criterion tends to select the correct number of classes, especially when the number of nodes is large.

8 $$\begin{aligned} \theta&=  (0.7, 0.3) , \end{aligned}$$

9 $$\begin{aligned} \pi&=  \left( \begin{array}{ccc} 0.8 &{} 0.2 \\ 0.3 &{} 0.9 \end{array} \right) , \alpha = \left( \begin{array}{ccc} 10.0 &{} 0.3 \\ 3.0 &{} 0.5 \end{array} \right) , \beta = \left( \begin{array}{ccc} 2.0 &{} 1.0 \\ 0.2 &{} 1.0 \end{array} \right) . \end{aligned}$$

**Table 1:** Convergence analysis of posterior class allocations and parameter estimates under the two-class model

*n*	$\sum _{i=1}^{n} I_{z_{i}=z_{i}^{*}} / n$	$||\theta - \theta ^{*}||_{2}$	$|| \pi - \pi ^{*} ||_{ \mathbb {F}}$	$||\alpha - \alpha ^{*}||_{\mathbb {F}}$	$|| \beta - \beta ^{*} ||_{\mathbb {F}}$
25	1	0.031	0.057	0.855	0.434
50	1	0.029	0.035	0.654	0.322
100	1	0.027	0.012	0.256	0.070
200	1	0.025	0.006	0.116	0.046
500	1	0.021	0.002	0.053	0.024

**Table 2:** Frequency of choosing *Q* blocks by ICL under different number of nodes *n* under the two-class model

*n* | *Q*	1	2	3	4	5
25	0	44	3	3	0
50	0	48	2	0	0
100	0	50	0	0	0
200	0	50	0	0	0
500	0	50	0	0	0

### Experiment 2 (three-class model)

50 network realizations are obtained under the three-class model with parameter values given in (10 and 11) for a range of values *n*.

10 $$\begin{aligned} \theta&=  (0.5,0.3,0.2) \end{aligned}$$

11 $$\begin{aligned} \pi&=  \left( \begin{array}{ccc} 0.60 &{} 0.20 &{} 0.30 \\ 0.30 &{} 0.90 &{} 0.10 \\ 0.60 &{} 0.50 &{} 0.20 \\ \end{array} \right) , \alpha = \left( \begin{array}{ccc} 0.50 &{} 2.00 &{} 1.00 \\ 0.30 &{} 0.02 &{} 6.00 \\ 2.00 &{} 0.05 &{} 3.00 \\ \end{array} \right) , \beta = \left( \begin{array}{ccc} 5.00 &{} 0.40 &{} 5.00 \\ 3.00 &{} 12.00 &{} 0.70 \\ 6.00 &{} 0.20 &{} 0.60 \end{array} \right) . \end{aligned}$$

We can see from Table 3 that the estimated parameters converge to their true values quickly as the number of nodes increases. The ICL criterion tends to overestimate the number of classes when the number of nodes is small, but consistently selects the correct model when the number of nodes is large (Table 4).

**Table 3:** Convergence analysis of posterior class allocations and parameter estimates under the three-class model

*n*	$\sum _{i=1}^{n} I_{z_{i}=z_{i}^{*}} / n$	$||\theta - \theta ^{*}||_{2}$	$|| \pi - \pi ^{*} ||_{\mathbb {F}}$	$||\alpha - \alpha ^{*}||_{\mathbb {F}}$	$|| \beta - \beta ^{*} ||_{\mathbb {F}}$
25	0.961	0.116	0.178	7.86	136.72
50	1	0.039	0.039	1.256	7.866
100	1	0.033	0.026	0.481	1.426
200	1	0.014	0.024	0.228	1.011
500	1	0.003	0.006	0.197	0.222

**Table 4:** Frequency of choosing *Q* blocks by ICL under different number of nodes *n* under the three-block model

*n*|*Q*	1	2	3	4	5
25	0	3	37	8	2
50	0	0	43	7	0
100	0	0	50	0	0
200	0	0	49	1	0
500	0	0	50	0	0

### Computational complexity

**Fig. 1:**
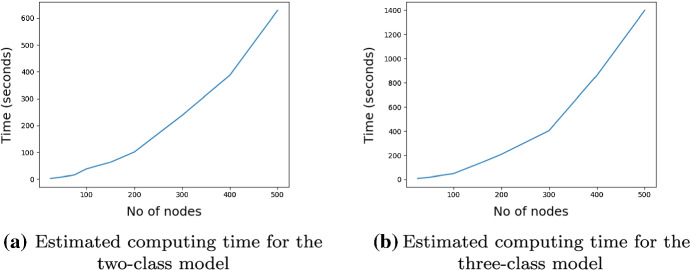
Estimated computing time for the two-class and three-class models

The computational complexity of the variational estimators derived in Sect. 5 scales as ${{\mathcal {O}}}(n^2)$, which has the same complexity as the variational algorithm developed by Daudin et al. (2008). Therefore, the algorithm may be prohibitively expensive for networks with more than 1000 nodes. The estimated computational time for the two-class model in Sect. 7.1 and for the three-class model in Sect. 7.2 for various values of *n* are shown in Fig. 1. The estimated computing time at each *n* is the average running time of the variational algorithm over 20 replications.

## Application: Washington bike data set

We apply the WSBM to analyse the Washington bike sharing scheme data set.^1^ Information with respect to start stations and end stations of trips as well as length (travel time) of trips are available in the data set. We select a time window of one week staring from January 10th, 2016 and construct the adjacency matrix *X* and weight matrix *Y* as follows:

- $X_{ij} = 1$ if there is trip starting from station *i* and finishing at station *j*.
- $Y_{ij}$ is the total length of trips (in minutes) from station *i* to station *j*.

**Fig. 2:**
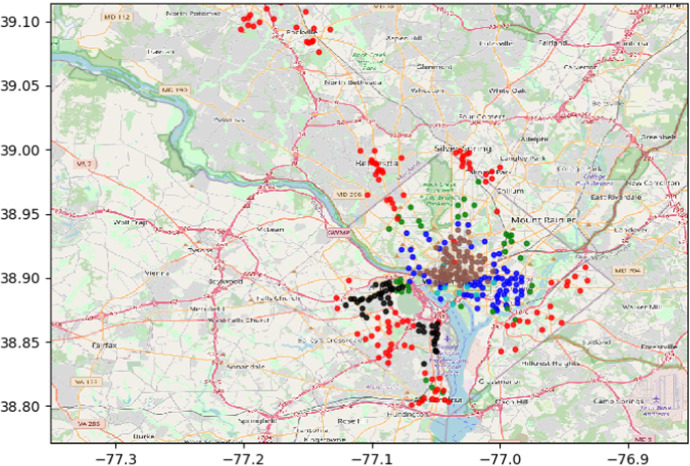
Bike stations. Class 1: blue. Class 2: green. Class 3: red. Class 4: cyan. Class 5: black. Class 6: brown (color figure online)

The resulting network consists of 370 nodes with an average out-degree of 36.14. The average total length of trips between any pair of stations is 42.15 minutes. We apply the ICL criterion to select the number of classes for the WSBM. For each number of classes *Q*, the variational inference algorithm is fitted to the network 20 times with 20 random initializations and the highest value of ICL is recorded, and the six-class model is chosen (Table 5). Each bike station is plotted on the map in Fig. 2 where its colour represents the estimated class assignment. We observe that bike stations in class 6 (colored in brown) tend to be concentrated in the central area of Washington wheraas stations in class 3 (colored in red) tend to be located further from the center. Figure 2 shows some spatial effect in the class assignment of bike stations whereby stations that are close in distance tend to be in the same cluster, with the exception of class 3 (colored in red). One potential extension of the model is to take into account the spatial locations of the bike stations as covariates.

The estimated class proportions $\hat{\theta }$ shown in 12 indicate that class 3 has the largest number of stations whereas class 4 has the smallest number of stations. The estimated $\hat{\pi }$ shows that within class connectivity is generally higher compared to between class connectivity. We further observe that the connection probabilities between bike stations in class 1, 4, and 6 are substantially higher. Interestingly, we observe a near symmetry in the matrix $\hat{\pi }$ indicating that the probability of having a trip from a station in class *k* to another station in class *l* is similar with the probability of having a trip from class *l* to class *k*.

**Fig. 3:**
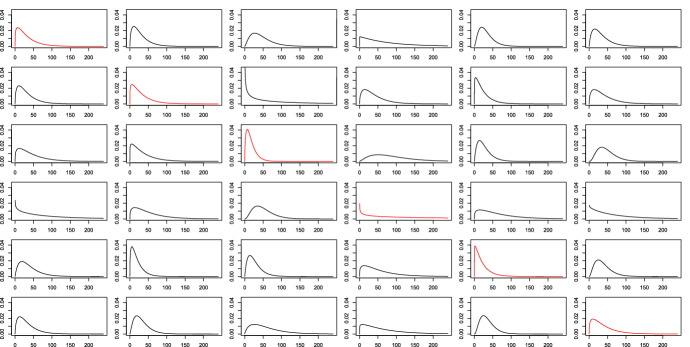
Estimated densities of total travel time between stations

The estimated densities of travel time between each pair of classes are shown in Fig. 3. We observe that the majority of the estimated densities have mode and mean close to 0, particularly for the estimated densities in the diagonal of Fig. 3. This implies that the total travel times between stations in the same class are quite short. In comparison, the total travel time between stations in different classes tend to be longer. This is reasonable as the distance between bike stations in different classes tend to be longer which in turn requires longer travel time.

**Table 5:** Model selection for the Washington Bike dataset using ICL criterion

*Q*	ICL
1	− 107157.24
2	− 93505.61
3	− 91688.33
4	− 90813.17
5	− 90300.90
6	− 89061.38
7	− 89326.35

12 $$\begin{aligned} \hat{\theta }&=  (0.1985, 0.0975, 0.3256, 0.0270, 0.1377, 0.2136) \end{aligned}$$

13 $$\begin{aligned} \hat{\pi }&=  \left( \begin{array}{cccccc} \mathbf {0.2847} &{} 0.0539 &{} 0.0036 &{} \mathbf {0.4268} &{} 0.0125 &{} \mathbf {0.2811} \\ 0.0594 &{} 0.0630 &{} 0.0172 &{} 0.1254 &{} 0.0156 &{} 0.0791 \\ 0.0049 &{} 0.0156 &{} 0.0185 &{} 0.0079 &{} 0.0092 &{} 0.0029 \\ \mathbf {0.4113} &{} 0.1140 &{} 0.0136 &{} \mathbf {0.8438} &{} 0.0532 &{} \mathbf {0.4494} \\ 0.0187 &{} 0.0318 &{} 0.0120 &{} 0.0910 &{} \mathbf {0.2159} &{} 0.0241 \\ \mathbf {0.2715} &{} 0.0514 &{} 0.0017 &{} \mathbf {0.4965} &{} 0.0159 &{} \mathbf {0.7268} \\ \end{array} \right) , \end{aligned}$$

## Discussion

This paper proposes a weighted stochastic block model (WSBM) for networks. The proposed model is an extension of the stochastic block model. A variational inference strategy is developed for parameter estimation. Asymptotic properties of maximum likelihood estimators and variational estimators are derived, and the problem of choosing the number of classes is addressed by using an ICL criteria. Simulation studies are conducted to evaluate the performance of variational estimators and the use of ICL to determine the number of classes. The proposed model and inference methods are an illustrative data set.

It is straightforward to extend the WSBM to allow node covariates. Let $w_i \in \mathbb {R}^{d}$ be the covariates for each node $i=1, \ldots , n$, and let $w_{ij}$ be the covariates for each pair of nodes, $i,j=1, \ldots , n, i \ne j$. The edge probability $p_{ij}$ between a pair of nodes *i*, *j* with node *i* in block *q* and node *j* in block *l* can be modelled as

$$\begin{aligned} \log \frac{p_{ij}}{1 - p_{ij}} = \xi _{ql,0} + \xi _{ql,1}^{T} w_{ij} + \xi _{ql,2}^{T} w_i + \xi _{ql,3}^{T} w_j, \end{aligned}$$

where $\xi _{ql,0} \in \mathbb {R}$ and $\xi _{ql,1}, \xi _{ql,2}, \xi _{ql,3} \in \mathbb {R}^{d}$. Conditional on block assignments and existence of an edge between a pair of nodes *i*, *j*, $Y_{ij}$ can be modelled as a Gamma random variable with mean $\mu _{ij}$ and variance $\sigma _{ij}$ where

$$\begin{aligned} \mu _{ij}&=  E(Y_{ij} | Z_i = q, Z_j = l, X_{ij} = 1) = \exp \big ( \phi _{ql, 0} + \phi _{ql, 1}^{T} w_{ij} + \phi _{ql, 2}^{T} w_i + \phi _{ql,3}^{T} w_j \big ), \\ \sigma _{ij}&=  Var( Z_i = q, Z_j = l, X_{ij} = 1) = \nu _{ql} \end{aligned}$$

where $$\phi _{ql,0} \in \mathbb {R}$$ and $$\phi _{ql,1}, \phi _{ql,2}, \phi _{ql,3} \in \mathbb {R}^{d}$$.

Many possible future extensions are possible. First, it is desirable to investigate further theoretical properties of maximum likelihood and variational estimators of WSBM parameters such as asymptotic normality of the estimators. Furthermore, some of the assumptions imposed in this work in order to ensure consistency of the estimators maybe relaxed. Moreover, the number of blocks is assumed to be fixed in the asymptotic analysis of the estimators. It would be interesting to allow the number of blocks to grows as the number of nodes grows.

## Auxiliary results

### **Definition 1**

A random variable *X* with mean $\mu = E(X)$ is sub-exponential if there are non-negative parameters $(\nu , b)$ such that

$$\begin{aligned} E(e^{t (X-\mu )}) \le e^{\frac{ \nu ^{2} t^{2} }{2} } \text{ for } \text{ all } |t| < \frac{1}{b} . \end{aligned}$$

The following lemma is a straight forward consequence of the definition.

### **Lemma 1**

*If the independent random variables* $\{ X_{i} \}_{i=1}^{n}$ *are sub-exponential with parameters* $( \nu _{i}, b_{i})$, $i=1,\ldots ,n$, *then* $\sum _{i=1}^{n} X_{i}$ *is sub-exponential with parameters* $(\sqrt{ \sum _{i=1}^{n} \nu _{i}^{2} }, \max _{i=1}^{n} b_{i} )$.

The following results show that for a Gamma random variable *Y* and a Bernoulli random variable *X*, both *XY* and $X \log Y$ are sub-exponential random variables. They are useful for the proof of the main theorems.

### **Proposition 3**

*If* $Y \sim Ga(a, b)$ *and* $X \sim Ber(\pi )$, *YX* *is a sub-exponential random variable.*

### *Proof*

The expectation of *YX* is given by

$$\begin{aligned} \mu := E(Y X) = \pi \frac{a}{b} , \end{aligned}$$

and thus

$$\begin{aligned} E(e^{t \mu }) = \exp \Big ( t \pi \frac{a}{b} \Big ) \text{. } \end{aligned}$$

The moment generating function of *YX* is given by

$$\begin{aligned} E(e^{t Y X}) = (1-\pi ) + \pi \Big (1-\frac{t}{b} \Big )^{-a} \end{aligned}$$

which is defined for $t < b$. Taylor series expansion of $(1 - \frac{t}{b})^{-a}$ around $t = 0$ gives that

$$\begin{aligned} \left( 1 - \frac{t}{b}\right) ^{-a} = 1 + \frac{a}{b} t + \frac{a (a+1)}{b^{2}} \frac{t^{2}}{2} + {{\mathcal {O}}}(t^{3}) \text{. } \end{aligned}$$

Similary, Taylor series expansion of $\exp \left( -t \frac{a}{b} \pi \right)$ around 0 gives that

$$\begin{aligned} \exp \left( -t \frac{a}{b} \pi \right) = 1 - \frac{a}{b} \pi t+ \frac{a (a+1)}{b^{2}} \pi \frac{t^{2}}{2} + {{\mathcal {O}}}(t^{3}) \text{. } \end{aligned}$$

Thus, we have

$$\begin{aligned} E(e^{t Y X})&=  1 - \pi + \pi + \frac{a}{b} \pi t + \frac{a (a+1)}{ b^{2}} \pi \frac{t^{2}}{2} + {{\mathcal {O}}}(t^{3}) \\&=  1+ \frac{a}{b} \pi t + \frac{a (a+1)}{b^{2}} \pi \frac{t^{2}}{2} + {{\mathcal {O}}}(t^{3}) . \end{aligned}$$

This leads to

$$\begin{aligned} E(e^{t (Y X - \mu )})&=  \Big (1+ \frac{a}{b} \pi t + \frac{a (a+1)}{ b^{2}} \pi \frac{t^{2}}{2} + {{\mathcal {O}}}(t^{3}) \Big ) \Big ( 1 - \frac{a}{b} \pi t+ \frac{a (a+1)}{b^{2}} \pi \frac{t^{2}}{2} + {\mathcal {O}}(t^{3}) \Big ) \\&=  1 + \frac{ (a^{2} + 2 a) \pi }{b^{2}} t^{2} + {{\mathcal {O}}}(t^{3}) \text{. } \end{aligned}$$

Hence, we can choose suitable $\nu , b$ such that

$$\begin{aligned} E(e^{t (Y X - \mu )}) \le \exp \Big ( \frac{\nu ^{2} t^{2}}{2} \Big ) \text{ for } \text{ all } |t| < \frac{1}{b} . \end{aligned}$$

$$\square $$

### **Proposition 4**

*If* $Y \sim Ga(a, b)$ *and* $X \sim Ber(\pi )$, $X \log Y$ *is a sub-exponential random variable.*

### *Proof*

The proof is analogous to the proof of Proposition 3. It is straightforward to show that

$$\begin{aligned} E( e^{t X \log Y}) = (1 - \pi ) + \pi \frac{ \varGamma ( \alpha + t) }{ \varGamma ( \alpha ) \beta ^t } \end{aligned}$$

and

$$\begin{aligned} \mu := E( X \log Y) = \pi ( \varPsi ( \alpha ) - \log (\beta ) ) \end{aligned}$$

where $\varPsi (\cdot )$ is the digamma function. The rest of the proof follows the same line as the proof of Proposition 3 by considering the Taylor series expansion of $e^{- \mu t}$ and $E( e^{t X \log Y})$ around $t= 0$. $\square$

The following lemma provides an upper bound for the tail probability of a sub-exponential random variable.

### **Lemma 2**

(Sub-exponential tail bound)*Suppose that* *X* *with mean* $\mu$ *is sub-exponential with parameters* $(\nu , b)$. *Then*

$$\begin{aligned} \mathbb {P}(X \ge \mu + t) \le \exp \Big [-\min \Big ( \frac{ t^{2} }{2 \nu ^{2}}, \frac{t}{2b} \Big ) \Big ] \text{. } \end{aligned}$$

The following inequality for suprema of random processes is needed for the proof of Theorem 2.

### **Proposition 5**

(Baraud 2010, Theorem 2.1) *Let* $(S(g))_{g \in {{\mathcal {G}}}}$ *be a family of real valued and centered random variables. Fix some* $g_{0}$ in ${{\mathcal {G}}}$.

*Suppose the following two conditions hold:*

*1.*: *There exist two arbitrary norms* $||\cdot ||_{2}$ *and* $||\cdot ||_{\infty }$ *on* ${{\mathcal {G}}}$ *and a nonnegative constant* *c* *such that for all* $g_{1}, g_{2} \in {{\mathcal {G}}} (g_{1} \ne g_{2})$, $$\begin{aligned} \mathbb {E} \Big [ e^{t (S(g_{1}) - S(g_{2}))} \Big ] \le \exp \Bigg ( \frac{t^{2} ||g_{1} - g_{2}||_{2}^{2} }{ 2(1-t c ||g_{1} - g_{2} ||_{\infty }) } \Bigg ) \end{aligned}$$ *for all* $t \in \Big [ 0, \frac{1}{c ||g_{1} - g_{2}||_{\infty }} \Big )$ .
*2.*: *Let* ${{\mathcal {S}}}$ *be a linear space with finite dimension* *D* *endowed with the two norms* $||\cdot ||_{\infty }$, $||\cdot ||_{2}$ *defined above, we assume that for constants* $u > 0$ *and* $b \ge 0$, $$\begin{aligned} {{\mathcal {G}}} \subset \{ g \in {{\mathcal {S}}} : ||g - g_{0}||_{\infty } \le u_1, c ||g-g_{0}||_{2} \le u_2 \} . \end{aligned}$$

*we have for all* $x>0$,

$$\begin{aligned} P \Big [ \sup _{g \in {{\mathcal {G}}}} | S(g) - S(g_{0}) | \ge \kappa ( \sqrt{ u^{2} (D+x) } + b(D+x) ) \Big ] \le 2 e^{-x} . \end{aligned}$$

*where* $$\kappa = 18$$.

## Proof

In this section, we prove Theorems 1, 2, 3 for the special case $\alpha _{q,l} = \alpha _{11}$ for all $(q,l) \in \{1, \ldots , Q \}$. The more general case can be proved similary by using the fact that $X_{ij} \log Y_{ij}$ is a sub-exponential random variable.

### Proof of Theorem 1

#### *Proof*

Our proof is adapted from Celisse et al. (2012). Using the fact that for every $z^{'}_{[n]} \in [z_{[n]}]$, $\mathbb {P}(z^{'}_{[n]} | X_{[n]}, Y_{[n]}) = \mathbb {P}(z_{[n]} | X_{[n]}, Y_{[n]})$, we have that

$$\begin{aligned} \mathbb {P}(Z_{[n]}&=  [z_{[n]}] | X_{[n]}, Y_{[n]}) = \sum _{z^{'}_{[n]} \in [z_{[n]}]} \mathbb {P}(z^{'}_{[n]} | X_{[n]}, Y_{[n]}) \\&=  |[z_{[n]}]| \mathbb {P}(z_{[n]} | X_{[n]}, Y_{[n]}) \end{aligned}$$

where $$ |[z_{[n]}]|$$ is the cardinality of the equivalence class $$ [z_{[n]}] $$.

By applying a similar approach as in ( Celisse et al. (2012); “Appendix B2”), one can derive an upper bound for $P^{*} \Bigg [ \sum _{ [z_{[n]}] \ne [z^{'}_{[n]}] } \frac{ \mathbb {P}( [ Z_{[n]} ] = [ z_{[n]} ] | X_{[n]}, Y_{[n]} )}{ \mathbb {P}( [ Z_{[n]} ] = [ z^{*}_{[n]} ] | X_{[n]}, Y_{[n]} ) } > t \Bigg ]$:

$$\begin{aligned}&P^{*} \Bigg [ \sum _{[z_{[n]}] \ne [z^{*}_{[n]}] } \frac{ \mathbb {P}([ z_{[n]} ] | X_{[n]}, Y_{[n]}) }{ \mathbb {P}( [z^{*}_{[n]}] | X_{[n]}, Y_{[n]}) }>t \Bigg ]&\\&\quad \le \sum _{r=1}^{n} \sum _{z_{[n]} \notin [z^{*}_{[n]}], ||z_{[n]} - z^{*}_{[n]} ||_{0} =r} P^{*} \Bigg [ \frac{ \mathbb {P}( z_{[n]} | X_{[n]}, Y_{[n]}) }{ \mathbb {P}( z^{*}_{[n]} | X_{[n]}, Y_{[n]}) } > \frac{t}{n^{r+1}(Q-1)^{r}} \Bigg ]&\end{aligned}$$

where $$||z_{[n]} - z^{*}_{[n]} ||_{0} $$ is the number of difference between $$z_{[n]}$$ and $$z^{*}_{[n]}$$.

To simplify notation, we write $$z := z_{[n]}$$, $$ X:=X_{[n]}$$, and $$Y=Y_{[n]}$$. We have that

$$\begin{aligned}&P^{*} \Bigg [ \frac{ \mathbb {P}( z | X, Y) }{ \mathbb {P}( z^{*} | X, Y) }> \frac{t}{n^{r+1}(Q-1)^{r}} \Bigg ] \\&\quad = P^{*} \Bigg [ \frac{ \mathbb {P}(Y | X, z) \mathbb {P}(X|z) \mathbb {P}(z)}{ \mathbb {P}(Y| X, z^{*}) \mathbb {P}( X|z^{*}) \mathbb {P}(z^{*})}> \frac{t}{n^{r+1}(Q-1)^{r}} \Bigg ]&\\&\quad = P^{*} \Bigg [ \log \frac{ \mathbb {P}(Y | X, z) }{ \mathbb {P}( Y | X, z^{*}) } + \log \frac{ \mathbb {P}(X | Z) \mathbb {P}(z) }{ \mathbb {P}(X|z^{*}) \mathbb {P}(z^{*}) }> \log \frac{t}{n^{r+1}(Q-1)^{r}} \Bigg ]&\\&\quad \le P^{*} \Bigg [ \Bigg | \log \frac{ \mathbb {P}(Y| X, z) }{ \mathbb {P}(Y|X,z^{*}) } - \mathbb {E}^{Z=z^{*}} \Big ( \log \frac{ \mathbb {P}(Y| X, z) }{ \mathbb {P}(Y|X,z^{*}) } \Big ) \Bigg |&\\&\quad> \frac{1}{2} \Big ( \log \frac{t}{n^{r+1}(Q-1)^{r}} - \mathbb {E}^{Z=z^{*}} \Big ( \log \frac{ \mathbb {P}(Y| X, z) }{ \mathbb {P}(Y|X,z^{*}) } \Big ) - \mathbb {E}^{Z=z^{*}} \Big ( \log \frac{\mathbb {P}(X|z) \mathbb {P}(z)}{ \mathbb {P}(X|z^{*}) \mathbb {P}(z^{*}) } \Big ) \Big ) \Bigg ]&\\&\qquad + P^{*} \Bigg [ \Big | \log \frac{\mathbb {P}(X|z) \mathbb {P}(z)}{ \mathbb {P}(X|z^{*}) \mathbb {P}(z^{*}) } - \mathbb {E}^{Z=z^{*}} \Big ( \log \frac{\mathbb {P}(X|z) \mathbb {P}(z)}{ \mathbb {P}(X|z^{*}) \mathbb {P}(z^{*}) } \Big ) \Big | \Bigg ]&\\&\quad > \frac{1}{2} \Big ( \log \frac{t}{n^{r+1}(Q-1)^{r}} - \mathbb {E}^{Z=z^{*}} \Big ( \log \frac{ \mathbb {P}(Y| X, z) }{ \mathbb {P}(Y|X,z^{*}) } \Big ) - \mathbb {E}^{Z=z^{*}} \Big ( \log \frac{\mathbb {P}(X|z) \mathbb {P}(z)}{ \mathbb {P}(X|z^{*}) \mathbb {P}(z^{*}) } \Big ) \Big ) \Bigg ]&\\&\quad =: I_{1} + I_{2}&\end{aligned}$$

Consider the first term on the RHS of the last inequality,

$$\begin{aligned}&\log \frac{\mathbb {P}(Y|X,z)}{\mathbb {P}(Y|X,z^{*})} - \mathbb {E}^{Z=z^{*}} \Big ( \log \frac{\mathbb {P}(Y|X,z)}{\mathbb {P}(Y|X,z^{*})} \Big )&\\&\quad = \sum _{i \ne j} \Big (\alpha _{11}^{*} \log \frac{\beta ^{*}_{z_{i},z_{j}}}{ \beta ^{*}_{z_{i}^{*},z_{j}^{*}} } \Big ) (X_{ij} - \pi ^{*}_{z_{i}^{*}, z_{j}^{*}} ) + ( \beta ^{*}_{z_{i}^{*}, z_{j}^{*}} - \beta ^{*}_{z_{i},z_{j}} ) \Big (Y_{ij} X_{ij} - \frac{\alpha _{11}^{*}}{\beta ^{*}_{z_{i}^{*},z_{j}^{*}} } \pi ^{*}_{z_{i}^{*},z_{j}^{*}} \Big )&\text{. } \end{aligned}$$

The summand in the summation above vanishes when $\beta ^{*}_{z_{i},z_{j}} = \beta ^{*}_{z_{i}^{*},z_{j}^{*}}$. For two vectors *z* and $z^{'}$, we define

$$\begin{aligned} D(z,z^{'}) = \{ (i,j) | i \ne j, \beta ^{*}_{z_{i},z_{j}} \ne \beta ^{*}_{z_{i}^{'},z_{j}^{'}} \} \end{aligned}$$

and let $$N_{r}(z) = | D(z,z^{*}) | $$ denote the number of terms in the summation. Set

$$\begin{aligned} s&=  \frac{1}{N_{r}(z)} \frac{1}{2} \Big \{ \log \frac{t}{n^{r+1}(Q-1)^{r}} - \mathbb {E}^{Z=z^{*}} \Big ( \log \frac{ \mathbb {P}(Y| X, z) }{ \mathbb {P}(Y|X,z^{*}) } \Big ) \\&- \mathbb {E}^{Z=z^{*}} \Big ( \log \frac{\mathbb {P}(X|z) \mathbb {P}(z)}{ \mathbb {P}(X|z^{*}) \mathbb {P}(z^{*}) } \Big ) \Big \} , \end{aligned}$$

one can show by Lemma B.3 of Celisse et al. (2012) that there exists some positive constant $c^{*}$ such that $s \ge c^{*} > 0$. We have that

$$\begin{aligned}&P^{*} \Bigg [ \frac{1}{N_{r}(z)} \Bigg | \log \frac{\mathbb {P}(Y|X,z)}{\mathbb {P}(Y|X,z^{*})} - \mathbb {E}^{Z=z^{*}} \Big ( \log \frac{\mathbb {P}(Y|X,z)}{\mathbb {P}(Y|X,z^{*})} \Big ) \Bigg |> 2s \Bigg ]&\\&\quad \le P^{*} \Bigg [ \frac{1}{N_{r}(z)} \Bigg | \sum _{i \ne j} \alpha _{11}^{*} \log \frac{ \beta ^{*}_{z_{i}, z_{j}} }{ \beta ^{*}_{z^{*}_{i},z^{*}_{j}} } (X_{ij} - \pi ^{*}_{z_{i}^{*},z_{j}^{*}}) \Bigg |> s \Bigg ]&\\&\qquad + P^{*} \Bigg [ \frac{1}{N_{r}(z)} \Bigg | \sum _{i \ne j} (\beta ^{*}_{z_{i}^{*}, z_{j}^{*}} - \beta ^{*}_{z_{i},z_{j}}) \Big (Y_{ij} X_{ij} - \frac{\alpha _{11}^{*}}{\beta ^{*}_{z_{i}^{*},z_{j}^{*}} } \pi ^{*}_{z_{i}^{*},z_{j}^{*}} \Big ) \Bigg | > s \Bigg ]&\end{aligned}$$

Assumption (A6) implies that the random variable $\alpha _{11}^{*} ( \log \beta ^{*}_{z_{i}, z_{j}} / \beta ^{*}_{z^{*}_{i},z^{*}_{j}} ) X_{ij}$ is bounded for all *i*, *j*. An application of Hoeffding’s inequality yields that

14 $$\begin{aligned} P^{*} \Bigg [ \frac{1}{N_{r}(z)} \Bigg | \sum _{i \ne j} \alpha _{11}^{*} \log \frac{ \beta ^{*}_{z_{i}, z_{j}} }{ \beta ^{*}_{z^{*}_{i},z^{*}_{j}} } (X_{ij} - \pi ^{*}_{z_{i}^{*},z_{j}^{*}}) \Bigg | > s \Bigg ] \le \exp \Bigg ( - \frac{N_{r}(z) s^{2}}{L_{1}} \Bigg ) \end{aligned}$$

for some constant $L_{1} > 0$.

By Proposition 3, the random variable $$Y_{ij} X_{ij} $$ is sub-exponential and the tail bound for sub-exponential random variables (Lemma 2) implies that

15 $$\begin{aligned}&P^{*} \Bigg [ \frac{1}{N_{r}(z)} \Bigg | \sum _{i \ne j} (\beta ^{*}_{z_{i}^{*}, z_{j}^{*}} - \beta ^{*}_{z_{i},z_{j}}) \Big (Y_{ij} X_{ij} - \frac{\alpha _{11}^{*}}{\beta ^{*}_{z_{i}^{*},z_{j}^{*}} } \pi ^{*}_{z_{i}^{*},z_{j}^{*}} \Big ) \Bigg | > s \Bigg ] \nonumber \\&\quad \le \max \bigg \{ \exp \bigg ( - \frac{ N_{r}(z) s^{2} }{L_{2}} \bigg ), \exp \bigg ( - \frac{N_{r}(z) s }{L_{3} } \bigg ) \bigg \} \end{aligned}$$

for some constants $L_{2}, L_{3} > 0$. Proposition B.4 of Celisse et al. (2012) shows that $N_{r}(z)$ is bounded below by

16 $$\begin{aligned} N_{r}(z) \ge \frac{\gamma ^{2}}{2} n || z_{[n]} - z^{*}_{[n]} ||_{0} \text{. } \end{aligned}$$

Combining inequalities (14), (15), (16), it is straight forward to show that

$$\begin{aligned} I_{1} =&{{\mathcal {O}}}(\exp ( -A_{1} n)) \text{. } \end{aligned}$$

By Theorem 3.1 of Celisse et al. (2012), we have

$$\begin{aligned} I_{2} =&{{\mathcal {O}}}( \exp (-A_{2}n) ) \end{aligned}$$

for some constant $A_{2}>0$. Therefore, with $A := \min \{ A_{1}, A_{2} \}$

$$\begin{aligned}&P^{*} \Bigg [ \sum _{[z_{[n]}] \ne [z^{*}_{[n]}] } \frac{ \mathbb {P}([ z_{[n]} ] | X_{[n]}, Y_{[n]}) }{ \mathbb {P}( [z^{*}_{[n]}] | X_{[n]}, Y_{[n]}) } >t \Bigg ] \\&\quad \le \sum _{r=1}^{n} {n \atopwithdelims ()r} (Q-1)^{r} {{\mathcal {O}}}(\exp (-An)) \\&\qquad \rightarrow 0 \end{aligned}$$

as $n \rightarrow \infty$. Since the upper bound does not depend on $z^{*}$, $P^{*}$ can be replaced by $\mathbb {P}$. $\square$

### Proof of Theorem 2

#### *Proof*

As the first step of the proof, we define the normalized complete data log-likelihood function

$$\begin{aligned}&\phi _{n}(z_{[n]}, \pi , \alpha , \beta ) = \frac{1}{n(n-1)} {\mathcal {L}}_{1}( X_{[n]}, Y_{[n]}; z_{[n]}, \pi , \alpha , \beta )&\\&\quad = \frac{1}{n(n-1)} \Big [ \sum _{i \ne j} \Big \{ X_{ij} \log \pi _{z_{i}, z_{j}} + (1-X_{ij}) \log (1-\pi _{z_{i},z_{j}}) \Big \}&\\&\qquad + X_{ij} \Big \{\alpha _{z_{i},z_{j}} \log \beta _{z_{i},z_{j}} + (\alpha _{z_{i},z_{j}} -1) \log Y_{ij} - \beta _{z_{i},z_{j}} Y_{ij} - \log \varGamma (\alpha _{z_{i},z_{j}}) \Big \} \Big ],&\end{aligned}$$

and its expectation

$$\begin{aligned}&\varPhi _{n}(z_{[n]}, \pi , \alpha , \beta ) = \mathbb {E} \Big [ \phi _{n}(z_{[n]}, \pi , \alpha , \beta ) \Big | Z_{[n]} = z_{[n]}^{*} \Big ]&\\&\quad = \frac{1}{n(n-1)} \bigg [ \sum _{i \ne j} \Big \{ \pi ^{*}_{z_{i}^{*}, z_{j}^{*}} \log \pi _{z_{i},z_{j}} + (1-\pi ^{*}_{z_{i}^{*},z_{j}^{*}}) \log ( 1 - \pi _{z_{i},z_{j}} ) \Big \}&\\&\qquad + \pi ^{*}_{z_{i}^{*},z_{j}^{*}} \bigg \{ \alpha _{z_{i},z_{j}} \log \beta _{z_{i},z_{j}} + (\alpha _{z_{i},z_{j}}-1) ( \log \beta ^{*}_{z_{i}^{*},z_{j}^{*}} + \psi (\alpha _{z^{*}_{i},z^{*}_{j}}^{*}) )&\\&\qquad - \beta _{z_{i},z_{j}} \frac{ \alpha _{z^{*}_{i},z^{*}_{j}}^{*}}{\beta ^{*}_{z_{i}^{*},z_{j}^{*}}} - \log \varGamma (\alpha _{z_{i},z_{j}}) \bigg \} \bigg ] \text{. }&\end{aligned}$$

The following proposition shows that $\phi _{n}(z_{[n]}, \pi , \alpha , \beta )$ uniformly converges to $\varPhi _{n}(z_{[n]}, \pi , \alpha , \beta )$. This result is an extension of Proposition 3.5 of Celisse et al. (2012).

#### **Proposition 6**

*Under assumptions (A1), (A2), (A5), (A6), we have*

$$\begin{aligned} \sup _{{{\mathcal {P}}}} | \phi _{n}(z_{[n]}, \pi , \alpha , \beta ) - \varPhi _{n}(z_{[n]}, \pi , \alpha , \beta ) | \xrightarrow [n \rightarrow \infty ]{\mathbb {P}} 0 \end{aligned}$$

*where* $$ {{\mathcal {P}}} := \{ (z_{[n]}, \pi , \alpha , \beta ): {(A1)}, {(A2)}, {(A5)}, {(A6)} \} $$.

#### *Proof*

We have that

17 $$\begin{aligned}&| \phi _{n}(z_{[n]}, \pi , \alpha , \beta ) - \varPhi _{n}(z_{[n]}, \pi , \alpha , \beta ) |&\nonumber \\&\quad \le \rho _{n} \Big | \sum _{i \ne j} ( X_{ij} - \pi ^{*}_{z_{i}^{*},z_{j}^{*}} ) \Big ( \log \frac{\pi _{z_{i},z_{j}}}{1-\pi _{z_{i},z_{j}}} + \alpha _{z_i, z_j} \log \beta _{z_{i},z_{j}} - \log \varGamma (\alpha _{11}) \Big ) \Big |&\nonumber \\&\qquad + \rho _{n} \Big | \sum _{i \ne j} \Big ( X_{ij} Y_{ij} - \pi _{z_{i}^{*},z_{j}^{*}}^{*} \frac{\alpha _{z_i, z_j}^{*}}{\beta ^{*}_{z_{i}^{*},z_{j}^{*}}} \Big ) \beta _{z_{i},z_{j}} \Big |&\nonumber \\&\qquad + \rho _{n} \Big | \sum _{i \ne j} \left( X_{ij} \log Y_{ij} - \pi ^{*}_{z_{i}^{*},z_{j}^{*}} \log \beta ^{*}_{z_{i}^{*},z_{j}^{*}} - \pi ^{*}_{z_{i},z_{j}} \psi (\alpha _{z^{*}_i, z^{*}_j}^{*}) \right) (\alpha _{z_i, z_j}-1) \Big |&\end{aligned}$$

where $\rho _{n} = 1/n(n-1)$. Notice that under Assumption (A2) and (A6),

$$\begin{aligned} \varPhi _{n}(z_{[n]}, \pi , \alpha , \beta ) < + \infty \text{. } \end{aligned}$$

Therefore, by Proposition 3.5 of Celisse et al. (2012),

$$\begin{aligned} \sup _{ {{\mathcal {P}}} } \rho _{n} \Big | \sum _{i \ne j} (X_{ij} - \pi ^{*}_{z_{i}^{*},z_{j}^{*}} ) \Big ( \log \frac{\pi _{z_{i},z_{j}}}{1-\pi _{z_{i},z_{j}}} + \alpha _{z_i, z_j} \log \beta _{z_{i},z_{j}} - \log \varGamma (\alpha _{z_i, z_j}) \Big ) \Big | \xrightarrow [n \rightarrow \infty ]{\mathbb {P}} 0 \end{aligned}$$

To bound the second term on the RHS of the inequality 17, we apply Proposition 5 (Theorem 2.1 of Baraud (2010)). We first define

$$\begin{aligned} S_{n}(\beta ) = \sum _{i \ne j} \bigg (X_{ij} Y_{ij} - \pi ^{*}_{z^{*}_{i},z^{*}_{j}} \frac{\alpha _{z^{*}_i, z^{*}_j}^{*}}{\beta ^{*}_{z_{i}^{*},z_{j}^{*}}} \bigg ) \beta _{z_{i},z_{j}} \text{. } \end{aligned}$$

Now, for two parameters $\beta ^{(1)}$ and $\beta ^{(2)}$,

$$\begin{aligned} S_{n}(\beta ^{(1)}) - S_{n}(\beta ^{(2)})&=  \sum _{i \ne j} \bigg (X_{ij} Y_{ij} - \pi ^{*}_{z^{*}_{i},z^{*}_{j}} \frac{\alpha _{z^{*}_i, z^{*}_j}^{*}}{\beta ^{*}_{z_{i}^{*},z_{j}^{*}}} \bigg ) ( \beta ^{(1)}_{z_{i},z_{j}} - \beta ^{(2)}_{z_{i},z_{j}} ) \\&= \sum _{q, l} \sum _{i \ne j, z_{i}=q, z_{j}=l} \bigg (X_{ij} Y_{ij} - \pi ^{*}_{z^{*}_{i},z^{*}_{j}} \frac{\alpha _{z^{*}_i, z^{*}_j}^{*}}{\beta ^{*}_{z_{i}^{*},z_{j}^{*}}} \bigg ) (\beta _{ql}^{(1)} - \beta _{ql}^{(2)}) \text{. } \end{aligned}$$

Since $$X_{ij} Y_{ij} $$ is sub-exponential with parameters $$(\nu , b)$$, $$\sum _{i \ne j, z_{i} = q, z_{j}=l} X_{i} X_{j} $$ is sub-exponential with parameters $$(\sqrt{n_{ql}} \nu , b)$$ by Lemma 3, where $$n_{ql} = | \{ (i, j): z_{i} = q, z_{j} = l\} |$$. Therefore, define the norms

$$\begin{aligned} ||\beta ^{(1)} - \beta ^{(2)}||^{2}_{2}&= n^{2} \nu ^{2} \sum _{q,l} (\beta _{ql}^{(1)} - \beta _{ql}^{(2)} )^{2} \\ ||\beta ^{(1)} - \beta ^{(2)}||^{2}_{\infty }&=  \sum _{q,l} (\beta _{ql}^{(1)} - \beta _{ql}^{(2)} )^{2} \text{, } \end{aligned}$$

we have

$$\begin{aligned}&\mathbb {E} \Big [ \exp ( t ( S_{n}(\beta ^{(1)}) - S_{n}(\beta ^{(2)}) ) \Big ]&\\&\quad = \prod _{q,l} E \Big [ \exp \Big ( t( \beta _{ql}^{(1)} - \beta _{ql}^{(2)} ) \sum _{i \ne j, z_{i}=q, z_{j}=l} \Big (X_{ij} Y_{ij} - \pi ^{*}_{z^{*}_{i},z^{*}_{j}} \frac{\alpha _{z_i^{*}, z_j^{*}}^{*}}{\beta ^{*}_{z_{i}^{*},z_{j}^{*}}} \Big ) \Big ) \Big ]&\\&\quad \le \prod _{q,l} \exp \Big \{ \frac{ n_{ql} \nu ^{2} t^{2} (\beta _{ql}^{(1)} - \beta _{ql}^{(2)})^{2} }{2} \Big \}&\\&\quad \le \exp \Big \{ \frac{ n^{2} \nu ^{2} t^{2} (\beta _{ql}^{(1)} - \beta _{ql}^{(2)})^{2} }{2} \Big \}&\\&\quad = \exp \Big \{ \frac{t^{2} || \beta ^{(1)} - \beta ^{(2)} ||_{2}^{2} }{2} \Big \}&\\&\quad \le \exp \Big \{ \frac{t^{2} || \beta ^{(1)} - \beta ^{(2)} ||_{2}^{2} }{2(1-t c ||\beta ^{(1)} - \beta ^{(2)}||_{\infty })} \Big \}&\end{aligned}$$

for all

$$\begin{aligned} t \in \Big [0, \frac{1}{ c || \beta ^{(1)} - \beta ^{(2)} || } \Big ) \text{, } \end{aligned}$$

where *c* is some negative constant. Therefore, the first condition of Proposition 5 is satisfied.

Fix some $\beta ^{(0)}$, (A6) implies that there exist $u_{1} = {{\mathcal {O}}}(n)$ and $u_{2} = {{\mathcal {O}}}(1)$ such that $||\beta - \beta ^{(0)}||_{2} \le u_{1}$, and $||\beta - \beta ^{(0)}||_{2} \le u_{2}$. Therefore the second condition of Proposition 5 is also satisfied with $D = Q^{2}$ . Now, we have

18 $$\begin{aligned} P^{*}( \rho _{n} \sup _{\beta } |S_{n}(\beta )|> & {} \eta ) \le P^{*}( \rho _{n} \sup _{\beta } | S_{n}(\beta ) - S_{n}(\beta ^{(0)}) |> \frac{\eta }{2} ) \nonumber \\&+ P^{*}( \rho _{n} | S_{n}(\beta ^{(0)}) | > \frac{\eta }{2} ) \text{. } \end{aligned}$$

Since $\sum _{i \ne j} X_{ij} Y_{ij} \beta ^{(0)}_{z_{i},z_{j}}$ is a sub-exponential random variable with parameters $(n \nu , b)$, the second term on the RHS of inequality 18 can be bounded by

19 $$\begin{aligned} P^{*}( \rho _{n} | S_{n}(\beta ^{(0)}) | > \frac{\eta }{2} ) = {\mathcal {O}}( \exp (-n^{2}) ) \text{. } \end{aligned}$$

To bound the first term on the RHS of inequality 18, we introduce the set ${{\mathcal {P}}}z_{[n]} = \{ (\pi , \alpha , \beta ): (z_{[n]}, \pi , \alpha , \beta ) \in {{\mathcal {P}}} \}$ for every $z_{[n]}$, and define the event

$$\begin{aligned} \varOmega _{n}(z_{[n]}) = \Big \{ \sup _{ {{\mathcal {P}}}(z_{[n]}) } \rho _{n} | S_{n}(\beta ) - S_{n}(\beta ^{(0)}) | \le \rho _{n} \kappa \sqrt{ u^{2} (Q^{2}+x_{n}) } + \rho _{n} b(Q^{2}+x_{n}) \Big \} \text{. } \end{aligned}$$

We have

20 $$\begin{aligned} P^{*}( \varOmega _{n}(z_{[n]})^{c}) = 2 e^{-x_{n}} \end{aligned}$$

by Proposition 5. Combining 18, 19, and 20,

$$\begin{aligned}&P^{*} \Big [ \rho _{n} \Big | \sum _{i \ne j} \Big ( X_{ij} Y_{ij} - \pi ^{*}_{z^{*}_{i},z^{*}_{j}} \frac{\alpha _{z_{i}^{*},z_{j}}^{*}}{\beta ^{*}_{z_{i}^{*},z_{j}^{*}}} \Big ) \beta _{z_{i},z_{j}} \Big |> \eta \Big ]\\&\quad \le \sum _{z_{[n]}} P^{*} \Big [ \Big \{ \sup _{ {{\mathcal {P}}}(z_{[n]}) } \rho _{n} | S_{n}(\beta ) - S_{n}(\beta ^{(0)}) |> \frac{\eta }{2} \Big \} \Big ] + P^{*} \Big [ \rho _{n} |S_{n}(\beta _{0}) |> \frac{\eta }{2} \Big ] \\&\quad \le \sum _{z_{[n]}} P^{*} \Big [ \Big \{ \sup _{ {{\mathcal {P}}}(z_{[n]}) } \rho _{n} | S_{n}(\beta ) - S_{n}(\beta ^{(0)}) |> \frac{\eta }{2} \Big \} \cap \varOmega _{n}(z_{[n]}) \Big ] + \sum _{z_{[n]}} 2 e^{-x_{n}} + \sum _{z_{[n]}} {{\mathcal {O}}}(e^{-n^{2}}) \\&\quad \le \sum _{z_{[n]}} P^{*} \Big [ \rho _{n} \kappa \sqrt{u^{2} (D+x_{n})} + \rho _{n} b (D+x_{n}) > \frac{\eta }{2} \Big ] + \sum _{z_{[n]}} 2 e^{-x_{n}} + \sum _{z_{[n]}} {{\mathcal {O}}}(e^{-n^{2}}) \end{aligned}$$

Since $z_{[n]}$ belongs to a set of cardinality at most $Q^{n}$, by choosing $x_{n} = n \log (n)$, the three sums converge to 0.

By using the fact that $X_{ij} \log Y_{ij}$ is a sub-exponential random variable, we can similarly show that

$$\begin{aligned} P^{*} \Big [ \sup _{ {{\mathcal {P}}} } \rho _{n} \Big | \sum _{i \ne j} \left( X_{ij} \log Y_{ij} - \pi ^{*}_{z^{*}_{i},z^{*}_{j}} \log \beta ^{*}_{z_{i}^{*},z_{j}^{*}} - \pi ^{*}_{z^{*}_{i},z^{*}_{j}} \psi (\alpha _{z_i^{*}, z_j^{*}}^{*}) \right) (\alpha _{z_i,z_j}-1) \Big | > \eta \Big ] \xrightarrow [n \rightarrow \infty ]{\mathbb {P}} 0. \end{aligned}$$

Since the convergence is uniform with respect to $z_{[n]}^{*}$, $P^{*}$ can be replaced by $\mathbb {P}$ and the proof is completed. $\square$

Proposition 6 allows us to establish the following result concerning the convergence of the normalized log-likelihood function. Proposition 7 is an extension of Theorem 3.6 of Celisse et al. (2012) and allows us to establish the consistency of MLE of $(\pi , \alpha , \beta )$.

#### **Proposition 7**

*We assume that assumptions (A1), (A2), (A3), (A5), (A6) hold. For every* $( \theta , \pi , \alpha , \beta )$, *set*

$$\begin{aligned} M_{n}(\theta , \pi , \alpha , \beta ) = (n(n-1))^{-1} {\mathcal {L}}_{2}(Y_{[n]},X_{[n]};\theta , \pi , \alpha , \beta ) , \end{aligned}$$

*and*

$$\begin{aligned} \mathbb {M}(\pi , \alpha , \beta , {{\mathcal {A}}})&=  \sum _{q,l} \theta ^{*}_{q} \theta ^{*}_{l} \sum _{q^{'},l^{'}} a_{q,q^{'}} a_{l,l^{'}} \Big [ \pi ^{*}_{ql} \log \pi _{q^{'},l^{'}} + (1-\pi ^{*}_{ql}) \log (1-\pi _{q^{'},l^{'}}) \\& \quad+ \pi ^{*}_{ql} E_{\alpha _{ql}^{*}, \beta ^{*}_{ql}}\Big ( \log f(.; \alpha _{q^{'},l^{'}}, \beta _{q^{'},l^{'}}) \Big ) \Big ] \end{aligned}$$

*where*

$$\begin{aligned} {{\mathcal {A}}} = \{ A=(a_{q,l})_{1 \le q,l \le Q} | a_{q,l} \ge 0, \sum _{l=1}^{Q} a_{kl} = 1 \} . \end{aligned}$$

*Then for any* $\eta > 0$,

$$\begin{aligned}&\sup _{d((\pi , \alpha , \beta ), (\pi ^{*}, \alpha ^{*}, \beta ^{*})) \ge \eta } \mathbb {M}(\pi , \alpha , \beta ) < \mathbb {M} (\pi ^{*}, \alpha ^{*}, \beta ^{*}) \text{, }\\&\sup _{ \theta , \pi , \alpha , \beta } | M_{n}(\theta , \pi , \alpha , \beta ) - \mathbb {M}(\pi , \alpha , \beta ) | \xrightarrow [ n \rightarrow \infty ]{ \mathbb {P} } 0 . \end{aligned}$$

#### *Proof*

We define the following:

$$\begin{aligned}&\hat{z}_{[n]}(\pi , \alpha , \beta ) = \text{ argmax}_{z} \phi _{n}(z_{[n]}, \pi , \alpha , \beta ) \text{, }&\\&\tilde{z}_{[n]}( \pi , \alpha , \beta ) = \text{ argmax}_{z} \varPhi _{n}( z_{[n]}, \pi , \alpha , \beta ) \text{, }&\\&\overline{A}_{\pi , \alpha , \beta } = \text{ argmax}_{A \in {{\mathcal {A}}}} \mathbb {M}(\pi , \alpha , \beta , {{\mathcal {A}}}) \text{, }&\\&\mathbb {M}(\pi , \alpha , \beta ) = \mathbb {M}(\pi , \alpha , \beta , \overline{A}_{\pi , \alpha , \beta } ) \text{. }&\end{aligned}$$

By a similar reasoning as in the proof of Theorem 3.6 of Celisse et al. (2012), we can show that $\overline{A}_{\pi ^{*}, \alpha ^{*}, \beta ^{*}} = I_{Q}$ and is unique. To show that for all $\eta >0$, $\sup _{d((\pi , \alpha , \beta ), (\pi ^{*}, \alpha ^{*}, \beta ^{*})) \ge \eta } \mathbb {M}(\pi , \alpha , \beta ) < \mathbb {M}( \pi ^{*}, \alpha ^{*}, \beta ^{*})$, we let $( \overline{a}_{ql} )_{q,l}$ denote coefficients of $\overline{A}_{\pi , \alpha , \beta }$. We have that

$$\begin{aligned}&\mathbb {M}( \pi , \alpha , \beta ) - \mathbb {M}( \pi ^{*}, \alpha ^{*}, \beta ^{*}) \\&\quad = - \sum _{q,l} \theta ^{*}_{q} \theta ^{*}_{l} \sum _{q^{'},l^{'}} \overline{a}_{q,q^{'}} \overline{a}_{l,l^{'}} \{ \mathbb {KL}_{B}( \pi _{ql}^{*}, \pi _{q^{'},l^{'}}) + \pi _{ql}^{*} \mathbb {KL}_{G}( (\alpha _{ql}^{*}, \beta _{ql}^{*}), (\alpha _{q^{'}, l^{'}} \beta _{q^{'},l^{'}}) ) \} \text{, } \end{aligned}$$

where $\mathbb {KL}_{B}( p,q)$ denotes the Kullback-Leibler divergence between two bernoulli distributions $\text{ Ber }(p)$ and $\text{ Ber }(q)$, and $\mathbb {KL}_{G}((a_,b_{1}),(a_2,b_{2}))$ denotes the Kullback-Leibler divergence between two gamma distributions $\text{ Ga }(a_1,b_{1})$ and $\text{ Ga }(a_2,b_{2})$.

Since the set $\{ (\pi , \alpha , \beta ) | d( ( \pi , \alpha , \beta ), (\pi ^{*}, \alpha ^{*}, \beta ^{*}) ) \ge \eta ) \}$ is compact by assumptions, there exists $(\pi ^{0}, \alpha ^{0}, \beta ^{0}) \ne (\pi ^{*}, \alpha ^{*}, \beta ^{*} )$ such that

$$\begin{aligned}&\sup _{d((\pi , \alpha , \beta ), (\pi ^{*}, \alpha ^{*}, \beta ^{*})) \ge \eta } \mathbb {M}(\pi , \alpha , \beta ) - \mathbb {M} (\pi ^{*}, \alpha ^{*}, \beta ^{*}) \\&\quad = \mathbb {M}( \pi ^{0}, \alpha ^{0}, \beta ^{0} ) - \mathbb {M} (\pi ^{*}, \alpha ^{*}, \beta ^{*}) < 0 \text{. } \end{aligned}$$

Next we show that

$$\begin{aligned} \sup _{ \theta , \pi ,\alpha , \beta } | M_{n}( \theta , \pi , \alpha , \beta ) - \mathbb {M}(\pi , \alpha , \beta ) | \xrightarrow [n \rightarrow \infty ]{\mathbb {P}} 0 \text{. } \end{aligned}$$

We first have the following bound:

21 $$\begin{aligned} | M_{n}( \theta , \pi , \alpha , \beta ) - \mathbb {M}(\pi , \alpha , \beta ) | & \le  | M_{n}( \theta , \pi , \alpha , \beta ) - \phi _{n}( \hat{z}, \pi , \alpha , \beta ) | \nonumber \\& \quad+ | \phi _{n}( \hat{z}, \pi , \alpha , \beta ) - \varPhi _{n}( \tilde{z}, \pi , \alpha , \beta ) | \nonumber \\&\quad+ | \varPhi _{n}( \tilde{z}, \pi , \alpha , \beta ) - \mathbb {M}( \pi , \alpha , \beta ) | \text{. } \end{aligned}$$

We first consider the first term on the RHS of inequality (21):

22 $$\begin{aligned}&\sup _{\theta , \pi , \alpha , \beta } | M_{n}( \theta , \pi , \alpha , \beta ) - \phi _{n}( \hat{z}, \pi , \alpha , \beta ) | \nonumber&\\&\quad = \sup _{\theta , \pi , \alpha , \beta } \frac{ | {\mathcal {L}}_{2}(Y_{[n]}, X_{[n]}; \theta , \pi , \alpha , \beta ) - {\mathcal {L}}_{1}(Y_{[n]}, X_{[n]}; \hat{z}_{[n]}, \pi , \alpha , \beta ) | }{ n (n-1) } \nonumber&\\&\quad \le \frac{ \log (1/\gamma ) }{ n - 1} \xrightarrow [n \rightarrow \infty ]{} 0 \text{. }&\end{aligned}$$

Consider the second term on the RHS of inequality (21), if $\phi _{n}( \hat{z}, \pi , \alpha , \beta ) < \varPhi _{n}( \tilde{z}, \pi , \alpha , \beta )$, we have

$$\begin{aligned} | \phi _{n}( \hat{z}, \pi , \alpha , \beta ) - \varPhi _{n}( \tilde{z}, \pi , \alpha , \beta ) |&=  \varPhi _{n}( \tilde{z}, \pi , \alpha , \beta ) - \phi _{n}( \hat{z}, \pi , \alpha , \beta ) \\ & \le  \varPhi _{n}( \tilde{z}, \pi , \alpha , \beta ) - \phi _{n}( \tilde{z}, \pi , \alpha , \beta ) \text{. } \end{aligned}$$

On the other hand, if $\phi _{n}( \hat{z}, \pi , \alpha , \beta ) \ge \varPhi _{n}( \tilde{z}, \pi , \alpha , \beta )$,

$$\begin{aligned} | \phi _{n}( \hat{z}, \pi , \alpha , \beta ) - \varPhi _{n}( \tilde{z}, \pi , \alpha , \beta ) |&=  \phi _{n}( \tilde{z}, \pi , \alpha , \beta ) - \varPhi _{n}( \hat{z}, \pi , \alpha , \beta ) \\ & \le\phi _{n}( \hat{z}, \pi , \alpha , \beta ) - \varPhi _{n}( \hat{z}, \pi , \alpha , \beta ) \text{. } \end{aligned}$$

Therefore, Proposition 6 implies that

23 $$\begin{aligned} \sup _{ \pi , \alpha , \beta } | \phi _{n}( \hat{z}, \pi , \alpha , \beta ) - \varPhi _{n}( \tilde{z}, \pi , \alpha , \beta ) | \xrightarrow [n \rightarrow \infty ]{\mathbb {P}} 0 . \end{aligned}$$

Last, we notice that under our assumptions (A2) and (A6), and using the strong law of large number,

24 $$\begin{aligned} \sup _{\pi ,\alpha ,\beta } | \varPhi _{n}( \tilde{z}, \pi , \alpha , \beta ) - \mathbb {M}( \pi , \alpha , \beta ) | \xrightarrow [n \rightarrow \infty ]{\mathbb {P}} 0 . \end{aligned}$$

Combining (22), (23) and (24), we have the desired result. $\square$

The consistency of $(\hat{\pi }, \hat{\alpha }, \hat{\beta })$ follows from Proposition 7 and Theorem 3.4 of Celisse et al. (2012). $\square$

### Proof of Thereom 3

#### *Proof*

We denote $\hat{P}( Z_{[n]} = z_{[n]} | X_{[n]}, Y_{[n]})$ as the conditional distribution of $Z_{[n]}$ under the parameters $(\hat{\theta }, \hat{\pi }, \hat{\alpha }, \hat{\beta } )$. The following result is an extension of Proposition 3.8 of Celisse et al. (2012) and is needed to establish the consistency of $\hat{\theta }$.

#### **Proposition 8**

*Assume that assumptions (A1)-(A6) hold, and there exists estimators* $\hat{\pi }, \hat{\alpha }, \hat{\beta }$ *such that* $|| \hat{\pi } - \pi ||_{\infty } = o_{P}(v_{n})$, $|| \hat{\alpha } - \alpha ||_{\infty } = o_{P}(v_{n})$, $|| \hat{\beta } - \beta ||_{\infty } = o_{P}(v_{n})$, *with* $v_{n} = o( \sqrt{ \log n }/n)$. *Let also* $\hat{\theta }$ *denote any estimator of* $\theta ^{*}$. *Then for every* $\epsilon > 0$,

$$\begin{aligned} P^* \Bigg [ \sum _{ z_{[n]} \ne z^{*}_{[n]}} \frac{ \hat{P}( Z_{[n]} = z_{[n]} | X_{[n]}, Y_{[n]}) }{ \hat{P}(Z_{[n]} = z^{*}_{[n]} | X_{[n]}, Y_{[n]}) }> \epsilon \Bigg ] &\le \kappa _{1} n e^{ - \kappa _{2} \frac{ (\log n)^{2} }{ n v_{n}^{2} } } + \mathbb {P} [ || \hat{\pi } - \pi ^{*} ||_{\infty }> v_{n} ] \\&\quad+ \mathbb {P} [ || \hat{\alpha } - \alpha ^{*} ||_{\infty }> v_{n} ] \\&\quad+ \mathbb {P} [ || \hat{\beta } - \beta ^{*} ||_{\infty } > v_{n} ] \end{aligned}$$

*for* *n* *large enough, and for some constants* $\kappa _{1}, \kappa _{2} > 0$ *and*

$$\begin{aligned}&\log \Bigg ( \frac{ \hat{P}( Z_{[n]} = z_{[n]} | X_{[n]}, Y_{[n]}) }{ \hat{P}(Z_{[n]} = z^{*}_{[n]} | X_{[n]}, Y_{[n]}) } \Bigg )&\\&\quad = \log \Bigg ( \frac{ \hat{P}(X_{[n]} | Z_{[n]} = z_{[n]}) P( Z_{[n]} = z_{[n]} ) }{ \hat{P}(X_{[n]} | Z_{[n]} = z^{*}_{[n]}) P( Z_{[n]} = z^{*}_{[n]} ) } \Bigg ) + \log \Bigg ( \frac{ \hat{P}( Y_{[n]} | X_{[n]}, Z_{[n]} = z_{[n]}) }{ \hat{P}( Y_{[n]} | X_{[n]},Z_{[n]} = z^{*}_{[n]}) } \Bigg )&\\&\quad = \sum _{i \ne j} \Bigg \{ X_{ij} \log \Big ( \frac{ \hat{\pi }_{z_{i},z_{j}} }{ \hat{ \pi }_{ z^{*}_{i},z^{*}_{j} } } \Big ) + (1-X_{ij}) \log \Big ( \frac{ 1 - \hat{\pi }_{z_{i}, z_{j}} }{ 1 - \hat{\pi }_{z^{*}_{i},z^{*}_{j}} } \Big ) \Bigg \} + \sum _{i} \log \frac{ \hat{ \theta }_{z_{i}} }{ \hat{ \theta }_{z^{*}_{i}} }&\\&\qquad + \sum _{i \ne j} \Bigg \{ X_{ij} (\hat{\alpha }_{z_{i},z_{j}} \log \hat{\beta }_{z_{i},z_{j}} - \hat{\alpha }_{z^{*}_{i},z^{*}_{j}} \log \hat{\beta }_{z_{i}^{*},z_{j}^{*}} ) + (\hat{\alpha }_{z_{i},z_{j}} - \hat{\alpha }_{z^{*}_{i},z^{*}_{j}}) X_{ij} \log Y_{ij}&\\&\qquad + X_{ij} Y_{ij} ( \hat{\beta }_{z^{*}_{i},z^{*}_{j}} - \hat{\beta }_{z_{i},z_{j}} ) + X_{ij} ( \log \varGamma ( \hat{\alpha }_{z^{*}_{i},z^{*}_{j}} ) - \log \varGamma (\hat{\alpha }_{z_{i},z_{j}}) ) \Bigg \}&\\&\quad := T_{0} + T_{1}&. \end{aligned}$$

#### *Proof*

We can write

$$\begin{aligned} T_1&=  \sum _{D^{*} } \Bigg \{ \pi ^{*}_{z^{*}_{i}, z^{*}_{j}} \frac{ \alpha _{z^{*}_i, z^{*}_j}^{*} }{ \beta ^{*}_{z^{*}_{i}, z^{*}_{j} } } ( \beta ^{*}_{z^{*}_{i}, z^{*}_{j}} - \beta ^{*}_{z_{i}, z_{j}}) + \pi ^{*}_{z^{*}_{i},z^{*}_{j}} \alpha _{z_i, z_j}^{*} \log \frac{ \beta ^{*}_{z_{i}, z_{j}} }{ \beta ^{*}_{z^{*}_{i}, z^{*}_{j} } } \Bigg \} \\&\quad+ \sum _{ D^{*} }\Bigg \{ \Big ( X_{ij} Y_{ij} - \frac{ \alpha ^{*}_{z^{*}_i, z^{*}_j} }{ \beta ^{*}_{z_{i}^{*}, z_{j}^{*}} } \pi ^{*}_{z_{i}^{*},z_{j}^{*}} \Big ) ( \beta ^{*}_{z_{i}^{*}, z_{j}^{*}} - \hat{\beta }_{z_{i},z_{j}} ) + (X_{ij} - \pi ^{*}_{z_{i}^{*}, z_{j}^{*} }) \alpha _{z_i, z_j}^{*} \log \frac{ \beta ^{*}_{z_{i}, z_{j}} }{ \beta ^{*}_{z_{i}^{*}, z_{j}^{*} } } \Bigg \} \\&\quad+ \sum _{ \hat{D} \cup D^{*} } \Bigg \{ \frac{ \alpha _{z_i^{*}, z_j^{*}}^{*} }{ \beta ^{*}_{z_{i}^{*}, z_{j}^{*} } } \pi ^{*}_{z_{i}^{*}, z_{j}^{*} } ( \hat{ \beta }_{z_{i}^{*}, z_{j}^{*} } - \beta ^{*}_{z_{i}^{*}, z_{j}^{*} } ) + \frac{ \alpha _{z^{*}_i, z^{*}_j}^{*} }{ \beta ^{*}_{z^{*}_{i}, z^{*}_{j} } } \pi ^{*}_{z^{*}_{i}, z^{*}_{j}} ( \hat{ \beta }_{z_{i}, z_{j} } - \beta ^{*}_{z_{i}, z_{j} } ) \\&\quad+ \pi ^{*}_{z^{*}_{i},z^{*}_{j}} \bigg ( \hat{\alpha }_{z_i, z_j} \log \frac{ \hat{\beta }_{z_{i},z_{j}} }{ \hat{ \beta }_{z^{*}_{i}, z^{*}_{j} } } - \alpha _{z_i, z_j}^{*} \log \frac{ \beta ^{*}_{z_{i},z_{j}} }{ \beta ^{*}_{z^{*}_{i}, z^{*}_{j} } } \bigg ) \Bigg \} \\&\quad+ \sum _{ \hat{D} \cup D^{*} } \Bigg \{ (X_{ij} - \pi ^{*}_{z^{*}_{i},z^{*}_{j}}) \bigg ( \hat{\alpha }_{z_i, z_j} \log \frac{ \hat{\beta }_{z_{i},z_{j}} }{ \hat{ \beta }_{z^{*}_{i}, z^{*}_{j} } } - \alpha _{z_i, z_j}^{*} \log \frac{ \beta ^{*}_{z_{i},z_{j}} }{ \beta ^{*}_{z^{*}_{i}, z^{*}_{j} } } \bigg ) \Bigg \} \\&\quad+ \sum _{ \hat{D} \cup D^{*} } \Bigg \{ \bigg ( X_{ij} Y_{ij} - \frac{ \alpha _{z^{*}_i, z^{*}_j}^{*} }{ \beta ^{*}_{z^{*}_{i},z^{*}_{j}} } \pi ^{*}_{z^{*}_{i},z^{*}_{j}} \bigg ) ( \hat{\beta }_{z^{*}_{i},z^{*}_{j}} - \beta ^{*}_{z^{*}_{i},z^{*}_{j}}) \\&\quad+ \bigg ( X_{ij} Y_{ij} - \frac{ \alpha _{z^{*}_i, z^{*}_j}^{*} }{ \beta ^{*}_{z^{*}_{i}, z^{*}_{j} } } \pi ^{*}_{z^{*}_{i}, z^{*}_{j}} \bigg ) ( \beta ^{*}_{z_{i}, z_{j}} - \hat{\beta }_{z_{i}, z_{j}}) \Bigg \} \\&=:  T_{1,1} + T_{1,2} + T_{1,3} + T_{1,4} + T_{1,5} \end{aligned}$$

where

$$\begin{aligned} D^{*}:&=  \{ (i,j): i \ne j, \pi ^{*}_{z_i, z_j} \ne \pi ^{*}_{ z^{*}_i, z^{*}_j } \} ,\\ \hat{D}:&=  \{ (i,j): i \ne j, \hat{\pi }_{z_i, z_j} \ne \hat{\pi }_{ z^{*}_i, z^{*}_j } \} . \end{aligned}$$

By the proof of Proposition 3.8 of Celisse et al. (2012), we have

$$\begin{aligned}&P^{*} \Bigg [ \bigg \{ \sum _{ [z_{[n]}] \ne [z_{[n]}^{*}] } \frac{ \hat{P}(Z_{[n]} = z_{[n]}|X_{[n]},Y_{[n]}) }{ \hat{P}(Z_{[n]} = z_{[n]}^{*}|X_{[n]},Y_{[n]}) }> \epsilon \bigg \} \cap \varOmega _{n} \Bigg ]&\\&\quad \le \sum _{r=1}^{n} \sum _{ z_{[n]} \notin [z_{[n]}^{*}], ||z_{[n]} - z_{[n]}^{*}||_{0} = r } P^{*} \Bigg [ \bigg \{ \log \frac{ \hat{P}(Z_{[n]}=z_{[n]}|X_{[n]},Y_{[n]}) }{ \hat{P}(Z_{[n]}=z_{[n]}^{*}|X_{[n]},Y_{[n]}) }> -5r \log n \bigg \} \cap \varOmega _{n} \Bigg ]&\\&\quad = \sum _{r=1}^{n} \sum _{ z_{[n]} \notin [z_{[n]}^{*}], ||z_{[n]} - z_{[n]}^{*}||_{0} = r } P^{*}[ \{ T_{0} + T_{1} > -5r \log n \} \cap \varOmega _{n}] \text{. } \end{aligned}$$

We have that

$$\begin{aligned} P^{*} [ \{ T_0 + T_1> -5r \log n \} \cap \varOmega _{n} ] \le P^{*}[ \{ T_0> -10r \log n \} \cap \varOmega _{n} \} + P^{*}[ \{ T_1 > 5r \log n \} \cap \varOmega _{n} ] \text{. } \end{aligned}$$

The proof of Proposition 3.8 of Celisse et al. (2012) shows that

25 $$\begin{aligned} P^{*}[ \{ T_0 > -10r \log n \} \cap \varOmega _{n} \} \le C_{1} \bigg \{\exp \bigg ( 8 n \log n - C_{2} \frac{ (\log n )^{2} }{n v_{n}^{2} } \bigg ) \bigg \}^{r} \text{. } \end{aligned}$$

We have that

$$\begin{aligned} T_{1,1}&=  \sum _{D^{*} } \pi ^{*}_{z^{*}_{i}, z^{*}_{j}} \frac{ \alpha _{z^{*}_i, z^{*}_j}^{*} }{ \beta ^{*}_{z^{*}_{i}, z^{*}_{j} } } ( \beta ^{*}_{z^{*}_{i}, z^{*}_{j}} - \beta ^{*}_{z_{i}, z_{j}}) + \pi ^{*}_{z^{*}_{i},z^{*}_{j}} \alpha _{11}^{*} \log \frac{ \beta ^{*}_{z_{i}, z_{j}} }{ \beta ^{*}_{z^{*}_{i}, z^{*}_{j} } } \\ & \le  |D^{*}| \max _{ (q,l) \ne (q^{'},l^{'}) } -\mathbb {KL}_{G}( (\alpha ^{*}_{ql}, \beta ^{*}_{ql})|| (\alpha ^{*}_{q^{'}, l^{'}} \beta ^{*}_{q^{'},l^{'}} ) ) \\& =  -|D^{*}| K^{*} \end{aligned}$$

Since $X_{ij} Y_{ij}$ is a sub-exponential random variable and $X_{ij}$ is bounded for all *i*, *j*, one can show that

26 $$\begin{aligned} P^{*}[T_{1,2} + \frac{1}{2} T_{1,1} > t] \le \max \bigg \{ \exp \big \{ -C_{1} \frac{ (t + |D^{*}|K^{*})^{2}}{|D^{*}|} \bigg \}, \exp \big \{ -C_{2} (t+|D^{*}|K^{*}\big \} \bigg \} \text{. } \end{aligned}$$

Using similar techniques as in the proof of Proposition 3.8 of Celisse et al. (2012), one can show that

27 $$\begin{aligned}&P^{*}[ \{ |T_{1,3}|> t \} \cap \varOmega _{n} ] \le P^{*} \bigg ( v_{n} > \frac{C_{3} t}{nr} \bigg ) \text{, } \end{aligned}$$

28 $$\begin{aligned}&P^{*}[ \{T_{1,4} > t \} \cap \varOmega _{n} ] \le \sum _{k} \sum _{D, |D|=k} Q^{2} \exp \bigg ( - \frac{ C_{4} t^{2} }{v^{2}_{n} (k+|D^{*}|)} \bigg ) \text{, } \end{aligned}$$

29 $$\begin{aligned}&P^{*}[T_{1,5} + \frac{1}{2} T_{1,1} > t]&\nonumber \\&\quad \le \sum _{k} \sum _{D, |D|=k} Q^{2} \max \Bigg \{ \exp \bigg [ - \frac{C_{5} (t + (|D^{*}|+k) K^{*})}{v_{n}} \bigg ],\nonumber \\&\qquad \exp \bigg [ - \frac{ C_{6} (t + (|D^{*}|+k) K^{*})^{2} }{ (|D^{*}| + k ) v_{n}^{2} } \bigg ] \Bigg \}&\text{. } \end{aligned}$$

Combining inequalities 25, 26, 27, 28, 29, and letting $v_{n} = o( \sqrt{ \log n}/ n)$, we have for some $B_{1}, B_{2}, B_{3} > 0$,

$$\begin{aligned}&P^{*} \Big [ \Big \{ \sum _{ [z_{[n]}] \ne [z_{[n]}^{*}]} \frac{ \hat{P}(z_{[n]} | X_{[n]}, Y_{[n]}) }{ \hat{P}(z_{[n]}^{*}|X_{[n]}, Y_{[n]}) } > \epsilon \Big \} \cap \varOmega _{n} \Big ]&\\&\quad \le \sum _{r=1}^{n} {n \atopwithdelims ()r} (Q-1)^{r} B_{1} \Big ( \exp \Big [ B_{2} n \log n - B_{3} \omega (n \log n) \Big ] \Big )^{r}&\\&\quad = B_{1} [(1+(Q-1) u_{n}^{'})^{n} - 1]&\end{aligned}$$

where $u_{n}^{'} = \exp \Big [ B_{2} n \log n - B_{3} \frac{ (\log n)^{2}}{n v_{n}^{2}} \Big ]$. Since $(1+(Q-1) u_{n}^{'})^{n} \rightarrow 1$ as $n \rightarrow \infty$, and the proof is completed. $\square$

The proof of the consistency of $\hat{\theta }$ is a consequence of the proposition above and follows the same lines as the proof of Theorem 3.9 of Celisse et al. (2012).$\square$

## Footnotes

1 Historical data available at https://www.capitalbikeshare.com/.
